# *p*-Phenylenediaminium iodide capping agent enabled self-healing perovskite solar cell

**DOI:** 10.1038/s41598-020-76365-y

**Published:** 2020-11-17

**Authors:** Parisa Zardari, Ali Rostami, Hemayat Shekaari

**Affiliations:** 1 Photonic and Nanocrystals Research Laboratory (PNRL), University of Tabriz, 5166614761 Tabriz, Iran; 2 SP-EPT Laboratory, Industrial Park of Advanced Technologies, ASEPE Company, 5364196795 Tabriz, Iran; 3 Department of Physical Chemistry, University of Tabriz, 5166616471 Tabriz, Iran

**Keywords:** Energy science and technology, Engineering, Materials science, Optics and photonics

## Abstract

In this study, *p*-Phenylenediaminium iodide (PDAI) is used to in-situ growth of 2D (PDA)_2_PbI_4_ perovskite layer between (FAPbI_3_)_0.85_(MAPbBr_3_)_0.15_ 3D perovskite and CuSCN as a cheap hole transport layer. The results indicate that the incorporation of 5 mg mL^−1^ PDAI leads to enlarged grain sizes, compact grain boundaries, reduced trap density, efficient charge extraction, and enhanced stability of perovskite film. Passivation of perovskite film with the appropriate amount of PDAI helps in achieving efficient perovskite solar cell with a PCE as high as 16.10%, a J_SC_ of 21.45 mA cm^−2^, a V_OC_ of 1.09 V, and FF of 70.21%, with negligible hysteresis and excellent moisture stability which remains 99.01% of its initial PCE value after 5 h in high relative humidity of 90 ± 5% and shows unchanged PCE after 1440 h in low relative humidity of 15 ± 5%. Most strikingly, this ultra-thin 2D passivation layer by the use of PDA cations as a bulky spacer not only passivates the defects on the surface of perovskite film but also induces self-healing properties in PSCs which can be rapidly recovered after keeping away from water vapor exposure. This study introduces the cheap and extra stable perovskite solar cells with outstanding self-healing ability towards commercialization.

## Introduction

Nowadays, organic–inorganic hybrid perovskite solar cells (PSCs) with fascinating optoelectronic properties have attracted remarkable attention in photovoltaic devices^1,2^. However, their instability under ambient conditions is one of the major limitations of practical implementation^3^. The second barrier in the commercialization of PSCs is the use of expensive materials as hole transport layer (HTL)^4^. The highest efficiency PSCs are developed based on mesoporous configuration, where used organic molecule 2,2′,7,7′-Tetrakis[N,N-di(4-methoxyphenyl)amino]-9,9′-spirobifluorene (spiro-OMeTAD) with dopants of lithium bis(trifluoromethane)sulfonyl)imide (Li-TFSI) and 4-tert-butylpyridine (TBP) as HTL. These materials not only destroy the device stability but also increase device cost^5^. Therefore, many studies have tried to find appropriate hole transport materials (HTMs) of improved stability and low cost^6–8^. CuSCN is one of the most promising inorganic HTMs^9^. CuSCN has a higher maximum hole mobility (0.01–0.1 cm^2^ V^−1^ s^−1^), which is far larger than of spiro-OMeTAD (6 × 10^–5^ cm^2^ V^−1^ s^−1^). Also, it has suitable energy levels as well as simplified synthesis routes as compared to spiro-OMeTAD^10^. Because of the streamlined and affordable procedures used to fabricate CuSCN HTMs, PSCs employing CuSCN HTMs instead of spiro-OMeTAD have a great opportunity for commercialization^11^. Despite the usefulness of CuSCN as HTL, there are two major problems with applying it. First, it has been reported that most solvents used for CuSCN deposition dissolve the perovskite materials^12^. Secondly, the perovskite layer has not been completely covered with the inorganic CuSCN film. The coated CuSCN is crystallized in a texture grain structure. So, the CuSCN HTL and perovskite interactions are insufficient^13^. Therefore, it is desirable to improve both the PCE and stability of PSCs from the viewpoint of interface engineering.

Mhaisalkar et al.^14^ have reported the formation of a 2D alkylammonium halide perovskite layer on the 3D film to passivate interfacial defects and vacancies. Also, an ultra-stable perovskite device has been introduced by engineering the (HOOC(CH_2_)_4_NH_3_)_2_PbI_4_ interface between MAPbI_3_ and spiro-OMeTAD with 14.6% PCE^15^. Bai et al.^16^ designed the 2D perovskite interface layer by in-situ growth of PEA_2_PbI_4_ on top of the 3D perovskite. This 2D passivating layer can induce larger Fermi-level splitting in the 2D-3D perovskite film under light illumination, leading to an improved V_OC_ and thus, an enhanced PCE^16^. In addition to ammonium salts with long-chain alkyl groups, different types of amines have also been used to form 2D perovskite interface layers on 3D perovskites. For example, benzylamine solution in chlorobenzene was poured on top of the 3D FAPbI_3_ lattice, leading to the 2D perovskite formation on FAPbI_3_. The modified device exhibited a champion efficiency of 19.2% and V_OC_ of 1.12 V and showed good stability in 50 ± 5 RH%^17^. By introducing these phenyl-groups or alkyl chains, the 2D networks are created, including inorganic perovskite layers of corner-sharing [PbX_6_]^4−^ octahedra restricted between interdigitating two ammonium cation layers. These two layers are combined by π–π or Van Der-Waals interactions between two alkyl or phenyl groups, which were belonging to a relatively week bonding. So, space between two [PbX_6_]^4−^ octahedra would be expanded due to the repulsive force^18,19^. As an alternative to these alkyl or phenyl groups, a suitable method is to use hydrophobic molecules like 5-Ammonium Valeric Acid (5-AVA) on the surface of perovskite grains via ionic and hydrogen bonds^20,21^. 2D perovskite used dialkylammonium cations as the bulky spacer has been synthesized by Lin and co-workers for the first time^19^. The two BA layers between the inorganic sheets of [PbI_6_]^4−^ octahedra have been replaced by a monolayer of DA cations. So, the replacement of van der Waals interactions to covalent -H_2_C-CH_2_- bond can be considered^19^.

Up to now, no studies have been reported on the use of *p*-phenyl-diammonium (PDA) groups as the bulky cations for constructing the 2D interface layer ((PDA)PbI_4_) between 3D perovskite and CuSCN layers. By using PDA groups, the inorganic sheets of [PbI_6_]^4−^ octahedral are held together by one molecule, meaning by ionic interactions. Therefore, the perovskite stability is increased as PbI_2_ is held firmly within the perovskite film, and the layered perovskite will be organized better^22,23^. We chose this barrier molecule due to (i) the diammonium functional group, which should provide a more organized structure; (ii) the relatively short length of the molecule, which will decrease the distance between the inorganic framework; (iii) the aromatic ring, which should have free π-electrons; therefore, it can enhance the charge transport and protect the surface of the perovskite by shielding it against reaction with atmospheric oxygen and water molecules^24^. Most strikingly, this ultra-thin 2D passivation layer by the use of PDA cations as a bulky spacer not only can passivate the defects of the perovskite surface but also induce self-healing properties in PSCs. The PSCs can return to its original phase and performance after exposure to water vapor and dried in ambient air, respectively.

## Results and discussion

**Figure 1:**
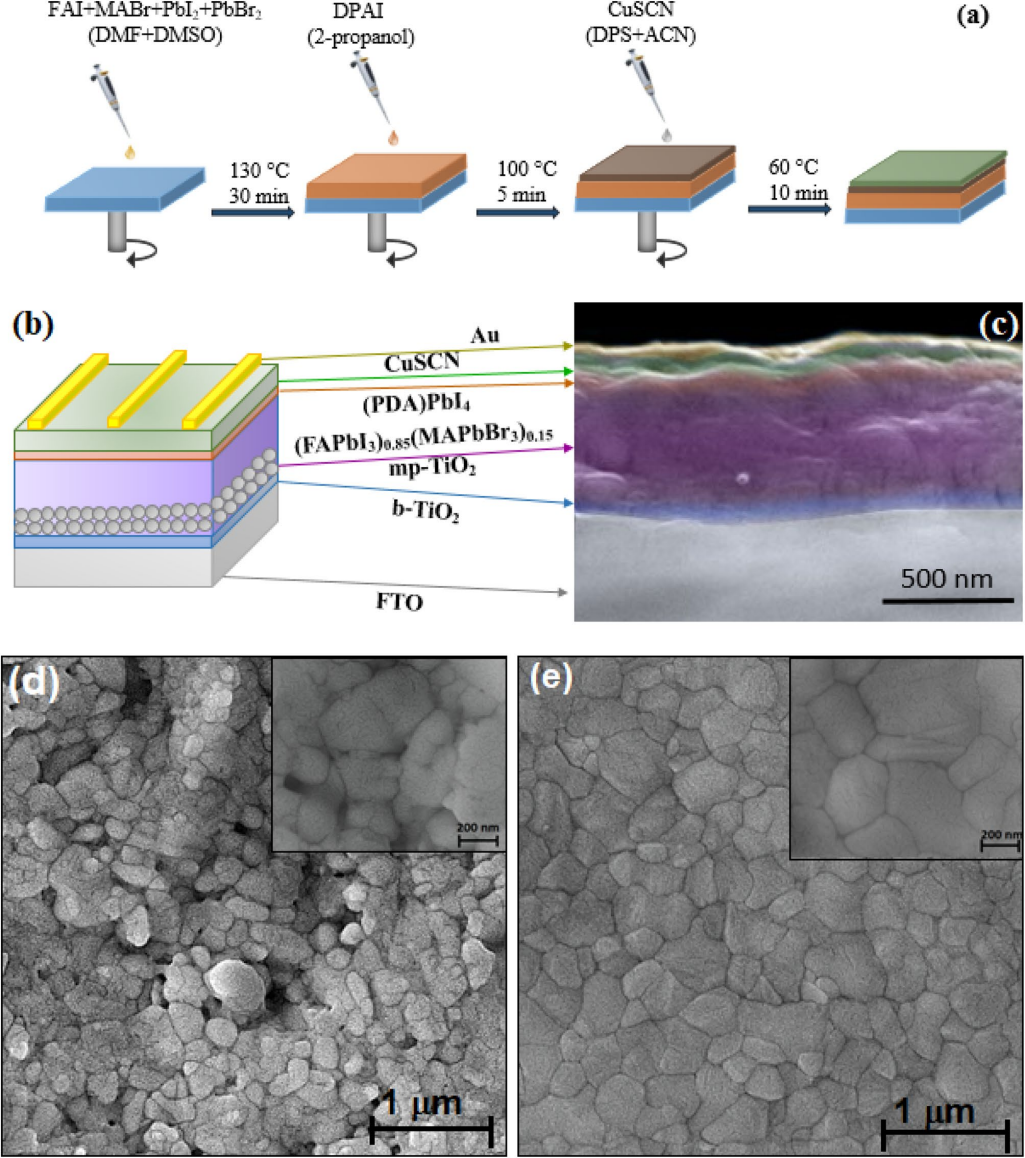
(**a**) Method to prepare perovskite solar cells with interface engineering. (**b**) Schematic device architecture used in the present work. (**c**) Cross-sectional SEM image of the complete device with interface engineering. (**d**) Low and high magnification of plane-view SEM images of perovskite films post-treated with 0 mg mL^−1^ and (**e**) 5 mg mL^−1^ of PDAI. Samples for SEM were prepared by deposition of perovskite films on mp-TiO_2_/bl-TiO_2_/FTO/glass.

Different concentrations of PDAI solutions (3–15 mg mL^−1^) was used to the in-situ growth of 2D perovskite at (FAPbI_3_)_0.85_(MAPbBr_3_)_0.15_ and CuSCN interface. The preparation scheme and the device configuration are illustrated in Fig. 1. The cross-sectional scanning electron microscopy (SEM) image is shown in Fig. 1c. In a 2D perovskite used one ammonium functional group as the long-chain organic cations, two ammonium can interact with each other by van der Waals force. So, space between two [PbX_6_]^4−^ octahedra would be expanded due to the repulsive force^18^. When a cation with two ammonium functional groups is used to prepare 2D perovskite, the inorganic frameworks are stack together by the stronger ionic bonds, as illustrated in Supplementary Fig. S1^19^. So, it is anticipated that the 2D perovskite will be formed as an ultra-thin layer, as shown in Fig. 1c ^25^. The SEM images of the perovskite films passivated by different concentrations of PDAI are illustrated in Fig. 1d and Supplementary Fig. S2. For the perovskite film without PDAI post-treatment, there are two separated phases (Fig. 1d). The dark grain region is perovskite crystal, and the bright grain phase is PbI_2_^26^. After post-treatment of perovskite film with 5 mg mL^−1^ of PDAI, the PbI_2_ layer has been fully covered (Fig. 1d)^27^_._ The surface of perovskite film passivated by PDAI is remarkably more compact compared with pristine film. When the solution concentration of PDAI reaches to 5 mg mL^−1^, there emerges a new layer that partly covered the (FAPbI_3_)_0.85_(MAPbBr_3_)_0.15_ film, which is speculated to be a 2D perovskite capping layer growth in the surface or interface of (FAPbI_3_)_0.85_(MAPbBr_3_)_0.15_ grain boundaries via reaction of PDAI and excessive PbI_2_^28^. Surprisingly, the (FAPbI_3_)_0.85_(MAPbBr_3_)_0.15_ film post-treated with 7 mg mL^−1^ of PDAI, changed into compact perovskite film with grains larger than 1 μm PDAI solution treatment (Supplementary Fig. S2b). So, PDAI solution can induce the pristine film second-growth into large grains with fewer grain boundary defects based on Ostwald ripening mechanism^29^. The use of higher PDAI concentrations adversely affects the perovskite morphology and the excessive PDAI is trapped in the GBs, which may be decomposed under illumination and also caused phase separation (Supplementary Fig. S2c,d)^30^.

**Figure 2:**
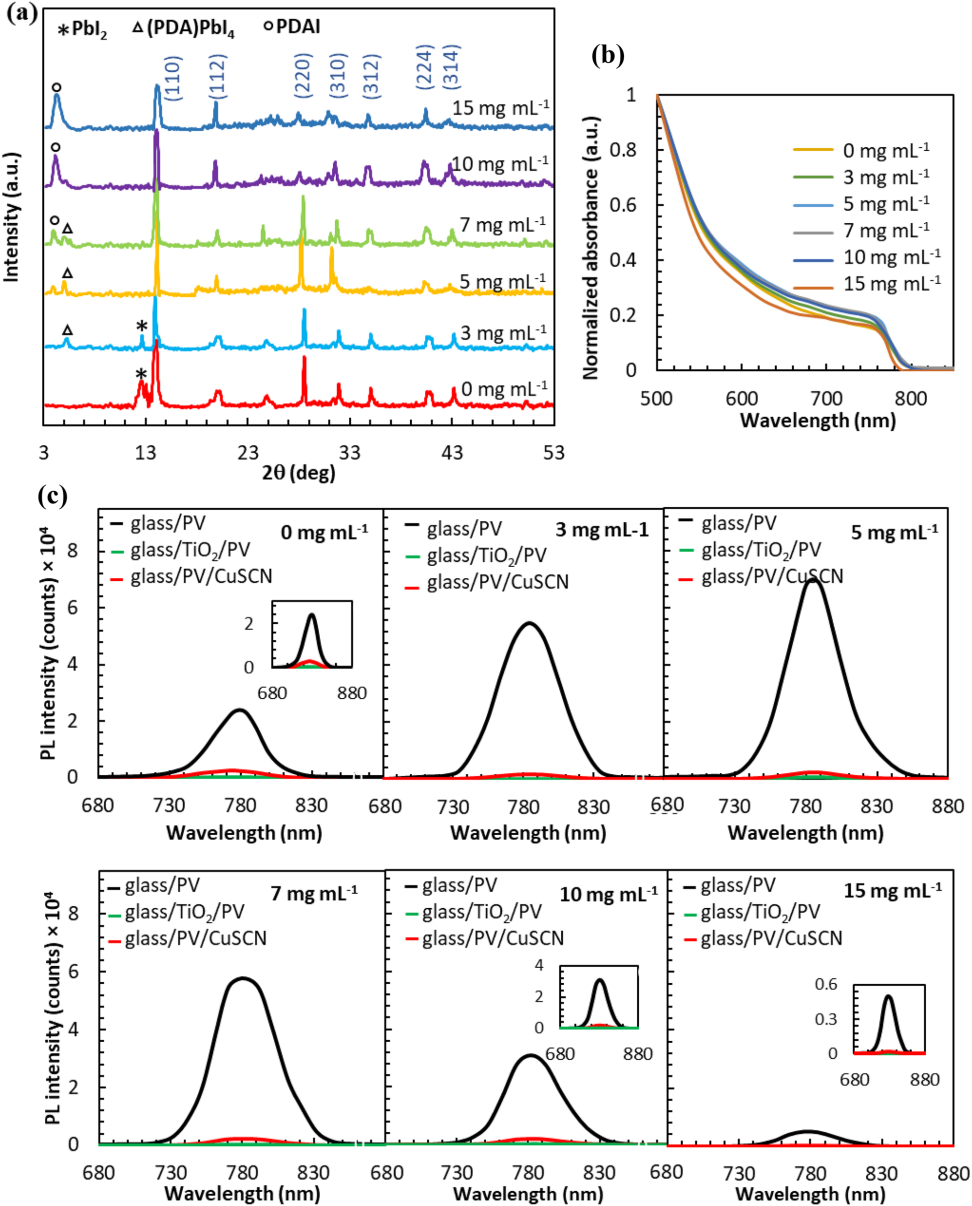
(**a**) XRD patterns and (**b**) UV–Vis absorption spectra of perovskite film post-treated with different concentrations of PDAI deposited on FTO/glass substrate. (**c**) Steady-state PL of perovskite films post-treated with different concentrations of PDAI with or without TiO_2_ and CuSCN layer.

The XRD patterns are utilized to investigate the effect of PDAI additive and 2D perovskite formation on the crystallinity of (FAPbI_3_)_0.85_(MAPbBr_3_)_0.15_ films (Fig. 2a). The XRD patterns show (FAPbI_3_)_0.85_ (MAPbBr_3_)_0.15_ crystal peaks containing three peaks at 14.2°, 28.4°, and 31.9°, related to (110), (220) and (310) crystal face, respectively^30^. Characteristic diffraction peak at 12.7° corresponding to PbI_2_ is observed in (FAPbI_3_)_0.85_(MAPbBr_3_)_0.15_ film due to the excess value of PbI_2_ in precursor composition^31^. Compared to pristine film, the intensity of the PbI_2_ peak is declined by the 3 mg mL^−1^ of PDAI, and a new peak at 5.1° is related to the 2D perovskite (PDA)PbI_4_. It means that the excess value of PbI_2_ in the pristine film is completely reacted with 3 mg mL^−1^ of PDAI and created 2D and quasi-2D perovskite. The XRD patterns of 2D perovskite film and PDAI powder are also shown in Supplementary Fig. S3. Furthermore, the characteristic peaks of (110), (220), (310) for perovskite films are notably enhanced by the addition of 5 mg mL^−1^ PDAI. The FWHM values of the characteristic (110) peak are presented in Supplementary Fig. S4. The smallest FWHM value belongs to the perovskite film passivated by 5 mg mL^−1^ PDAI, showing its excellent crystallization with a preferred orientation of (110)^30^. However, a new peak appeared at 3.9° in the presence of the higher concentration of PDAI is related to the diffraction pattern of PDAI powder.

Figure 2b indicates the normalized UV–Vis absorption spectra of perovskite films. A little improved absorbance is obtained for (FAPbI_3_)_0.85_(MAPbBr_3_)_0.15_/(PDA)PbI_4_ films prepared in the presence of PDAI near the bandgap absorption region. As the PDAI content is increased up to 5 mg mL^−1^, the perovskite film absorption is increased that is related to the PDAI addition impact on the preferred orientation crystal structure and uniform surface morphology^32^. When the PDAI addition amount is increased up to 15 mg mL^−1^, the absorbance is sharply decreased, which can relate to the crystallinity decrease, according to the SEM and XRD results. The bandgap of perovskite films can be deduced from the Tauc plot (Supplementary Fig. S5). The bandgap is calculated 1.55 eV for control pristine film and 1.54 eV for (FAPbI_3_)_0.85_(MAPbBr_3_)_0.15_/(PDA)PbI_4_ films prepared in the presence of 3–10 mg mL^−1^ PDAI. For (FAPbI_3_)_0.85_(MAPbBr_3_)_0.15_/(PDA)PbI_4_ film passivated by 15 mg mL^−1^, the bandgap is increased to 1.56 eV.

The photoluminescence (PL) spectra of the perovskite layer are shown in Fig. 2c, which shows a peak at 780 nm with the FWHM of about 50 nm^30^. The perovskite films passivated by 3, 5 and 7 mg mL^−1^ of PDAI indicate an enhanced PL yield in the comparison to the reference film, indicating less involvement in carrier recombination. On the other hand, the (FAPbI_3_)_0.85_(MAPbBr_3_)_0.15_ film post-treated with 5 mg mL^−1^ PDAI presents a higher fluorescence peak in comparison to the control film, suggesting the uniform, pinhole-free, reduced density of GBs and compact structure of perovskite resulted in low density of charge-trapping and recombination sites as illustrated in the SEM results^33,34^. It is reported that the charge recombination is minimized in the presence of ammonium cations which have benzene ring in their structure. It is due to prolonging the electron recombination because of the carrier accumulation in the benzene ring^35^. In the presence of 10 and 15 mg mL^−1^ PDAI, the large density of unreacted PDAI and relatively lose GBs leads to light scattering and excessive charge-trapping site creation^36,37^. Besides, the PL peak intensity of glass/perovskite/CuSCN samples is significantly decreased in the comparison of glass/perovskite ones, which can be related to the suitable valence band position of the HTMs over perovskite layer. In other words, the extra reduction in the PL spectrum is observed for glass/perovskite/CuSCN samples, where the 2D perovskite is inserted by in-situ growth as an interface. It is because of the better CuSCN coverage on 2D perovskite and interface engineering.

**Figure 3:**
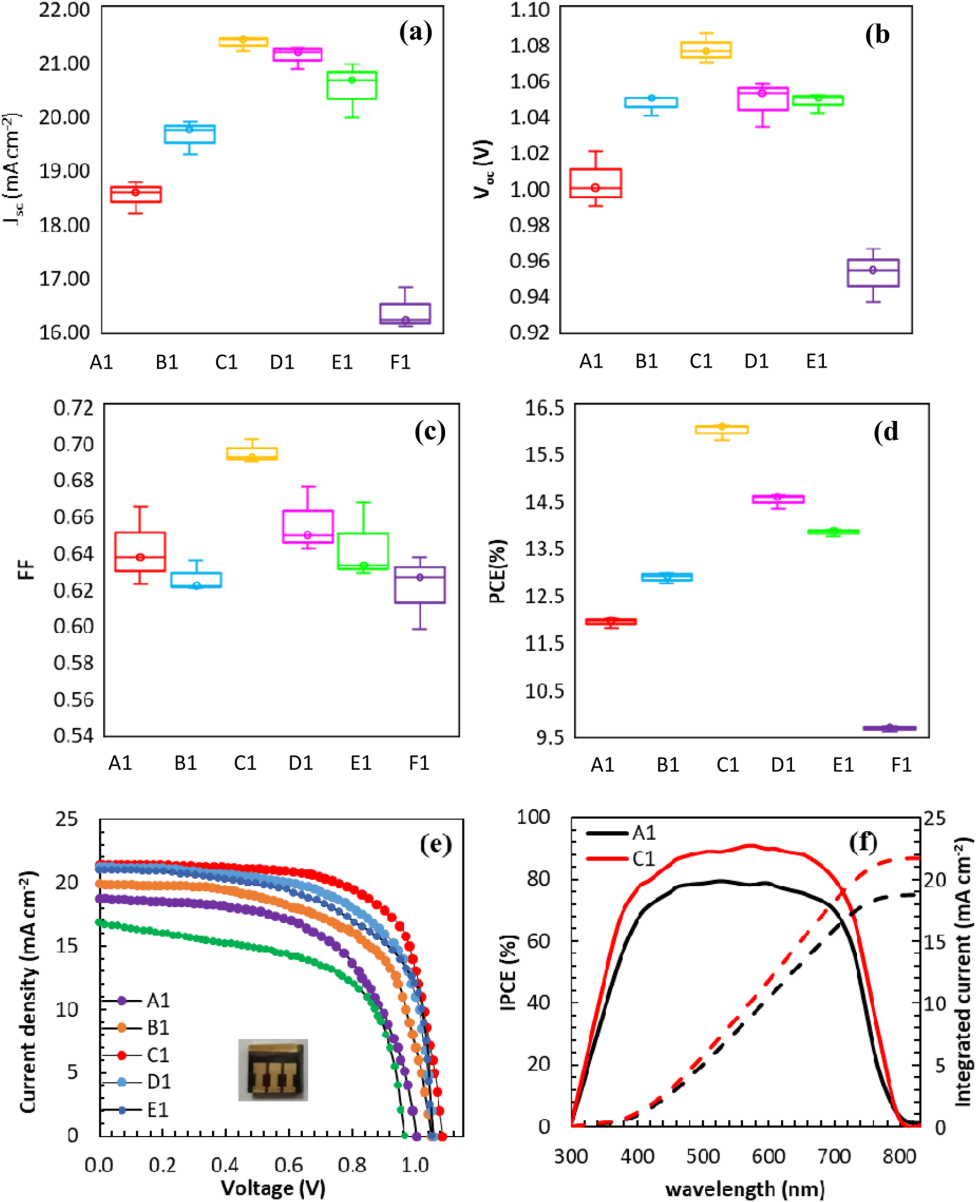
Statistic (**a**) J_SC_, (**b**) V_OC_, (**c**) FF, and (**d**) PCE of devices A1–F1. (**e**) J–V curves of devices measured in reverse scan at a scan rate of 50 mV s^−1^ under AM 1.5G. (**f**) The corresponding IPCE spectra of the best performing device C1 and controlled device A1 in which dashed lines stand for current density integrated from IPCE spectra.

In this study, twelve kinds of devices (A_1_ to F_1_ and A_2_ to F_2_) were manufacture and studied systematically. Devices are defined as follows. Device A: (FAPbI_3_)_0.85_(MAPbBr_3_)_0.15_ was used as an absorbent layer without in-situ growth of 2D perovskite on top of it. Device B to F: (FAPbI_3_)_0.85_(MAPbBr_3_)_0.15_ layer post-treated with different concentrations of PDAI (3, 5, 7, 10 and 15 mg mL^−1^) using spin-coating and in-situ growth of (PDA)PbI_4_ on top of the 3D perovskite layer. Devices A_1_ to F_1_ were fabricated by the spin-coating of CuSCN on top of the perovskite layer as HTL and devices A_2_ to F_2_ were fabricated without CuSCN HTL. Figure 3e shows the photocurrent density–voltage of the devices. All of the steps related to the preparation of solution precursor and spin-coating of the perovskite layer were performed at ambient conditions. The results indicate that J_SC_ and V_OC_ are increased by spin-coating of 3 mg mL^−1^ PDAI on the (FAPbI_3_)_0.85_(MAPbBr_3_)_0.15_ layer (Fig. 3a,b). The higher values of J_SC_ and V_OC_ for device B1 in the comparison of A1 are related to the low density of GBs, large grain size, and the pinhole-free surface of the absorbent layer because of the surface passivation. Furthermore, the enhancement of V_OC_ is due to the in-situ growth of (PDA)PbI_4_ as a 2D interface layer between (FAPbI_3_)_0.85_ (MAPbBr_3_)_0.15_ and CuSCN, which can induce a more effective interaction between absorbent layer and HTL, leading to accelerate the hole transfer to the upper layers by strong contact of perovskite and CuSCN^13^. Additionally, the different trend is obtained for FF change, that it is declined for device B1 and then increased for device C1 (Fig. 3c). It is reasonably assumed that the thickness of (PDA)PbI_4_ layer on the perovskite film surface depends on the PDAI amount in the precursor solution. In other words, (PDA)PbI_4_ film will be produced with inadequate thickness and incomplete coverage for a small quantity of PDAI (3 mg mL^−1^) in the precursor solution. So, the initial decrease of FF of device C1 might be related to the incomplete conversion of PbI_2_ into 2D (PDA)PbI_4_, which is the statement of the thickness and coverage importance for 2D perovskite^21^. By spin-coating of 5 mg mL^−1^ of PDAI solution on the 3D perovskite film, J_SC_, V_OC_ and FF values are increased, and the highest performance is achieved for device C1 with an optimal PCE of 16.10% (Fig. 3d), a J_SC_ of 21.45 mA cm^−2^, a V_OC_ of 1.09 V, and FF of 70.21%. The device C1 shows a champion efficiency of 17.52%, as shown in Supplementary Table S9.

Our results are somewhat different from the similar report on surface modification of (FAPbI_3_)_0.88_(CsPbBr_3_)_0.12_ with 5-AVAI, which was reported a PCE of 16.75%, a J_SC_ of 21.93 mA cm^−2^ and a V_OC_ of 1.068 V for champion cell^21^. The higher value of PCE and J_SC_ might be related to different perovskite composition. Contrary, the obtained V_OC_ is greater for our study.

The F1 device shows a decreased PCE of 9.60%. The unreacted diammonium can be trapped in the GBs of perovskite films and make adversely impact on the device performance. Also, the excess value of PDAI can increase series resistance, resulting in a decline of J_SC_, V_OC_ and PCE.

The IPCE spectra of devices A1 and C1 are summarized in Fig. 3f. The device C1 indicates higher IPCE than A1 between 350 and 750 nm. It is interesting that the absorption range of devices is almost the same, which points out that the 2D perovskite interface layer does not cause a significant change in the (FAPbI_3_)_0.85_(MAPbBr_3_)_0.15_ bandgap. The integrated currents of devices A1 and C1 are 18.77 and 21.73 mA cm^−2^, respectively, which are in good agreement with the corresponding values in Fig. 3a.

J-V hysteresis in PSCs is an important issue since it is related to the stability^38^. The causes of hysteresis have been reviewed as follows: Charge trapping/de-trapping related to the surface defects and corresponding density at the interfaces^39^, Ferroelectric polarization in perovskite materials^40^ and ion migration^41^. Accordingly, the J–V curves of devices under different scan directions were recorded after 30 days and shown in Supplementary Fig. S7. Devices were stored at ambient atmosphere (15 ± 5% RH) in the dark. The corresponding parameters of the devices are inserted in Table 1. The hysteresis index (HI) is calculated as Eq. (1)^42^:

1 $${\text{HI}} = \left[ {{\text{J}}_{{{\text{RS}}}} \left( {0.{\text{8 V}}_{{{\text{OC}}}} } \right) - {\text{J}}_{{{\text{FS}}}} \left( {0.{\text{8 V}}_{{{\text{OC}}}} } \right)} \right]/{\text{J}}_{{{\text{RS}}}} \left( {0.{\text{8 V}}_{{{\text{OC}}}} } \right)$$

**Table 1:** Photovoltaic performance parameters and calculated hysteresis index of devices based on different concentrations of PDAI post-treatment, with different scanning directions, after one-month maintenance in the ambient condition with 15 ± 5% RH and dark.

Device	J_SC_ [mA cm^−2^]		V_OC_ [V]		FF		PCE [%]		HI
	FS	RS	FS	RS	FS	RS	FS	RS	
A1	19.41	18.63	1	0.96	0.48	0.47	9.26	8.48	− 0.219
B1	20.66	20.02	1.04	1.02	0.56	0.51	12.02	10.4	− 0.205
C1	21.92	22.01	1.06	1.1	0.68	0.66	15.81	15.99	− 0.027
D1	21.24	21.45	1.03	1.07	0.57	0.58	12.56	13.41	0.036
E1	20.35	20.02	1.07	1.025	0.59	0.63	12.94	12.85	− 0.044
F1	17.3	17	0.98	0.936	0.54	0.56	9.2	8.93	0.049

where J_FS_ (0.8 V_OC_) and J_RS_ (0.8 V_OC_) show current density at 80% of V_OC_ for the forward and reverse scan, respectively. Devices A1 (HI = − 0.219) and B1 (HI = − 205) show similar hysteresis. Significantly reduced hysteresis is obtained for device C1 (HI = − 0.027) in comparison to devices A1 and B1, which is attributed to the decrease of the defect and trap sites by PDAI passivation and 2D layer formation as showed by SEM and XRD results. By spin-coating of the optimum value of PDAI (5 mg mL^−1^) on pristine film, more compact (FAPbI_3_)_0.85_(MAPbBr_3_)_0.15_ film with a lower density of GBs is achieved. It is reported that defects are made by vacancies in perovskite. The applied external electric field can create a driving force for ions and vacancies, especially through the GBs. These ions and vacancies can be accumulated at the perovskite-HTL and ETL interface, leading to interrupt the photo-generated electron and hole extraction due to increased capacitance. So, GB engineering is a key issue in hysteresis reduction^38^. It is reported that the passivation agents spun on the pristine film can penetrate the perovskite film via GBs^43^. So, PDAI can passive the iodide-rich trap sites at the GBs and suppress iodide ion migration to the interfaces under applied bias. Also, 2D perovskite ((PDAI)PbI_4_) will be able to decrease hysteresis through the effective physical barrier providing high activation energy for diffusion to hinder the ion migration and charge accumulation near the perovskite/HTL junctions^44^. It indicates that PDAI not only passivates the GBs in bulk perovskite but also cause to more compact film with a lower density of GBs. So, it can suppress the charges and ions accumulations at interfaces and prevents interface electrode polarization. So, interfacial and also bulk perovskite engineering is both supposed by PDAI.

**Figure 4:**
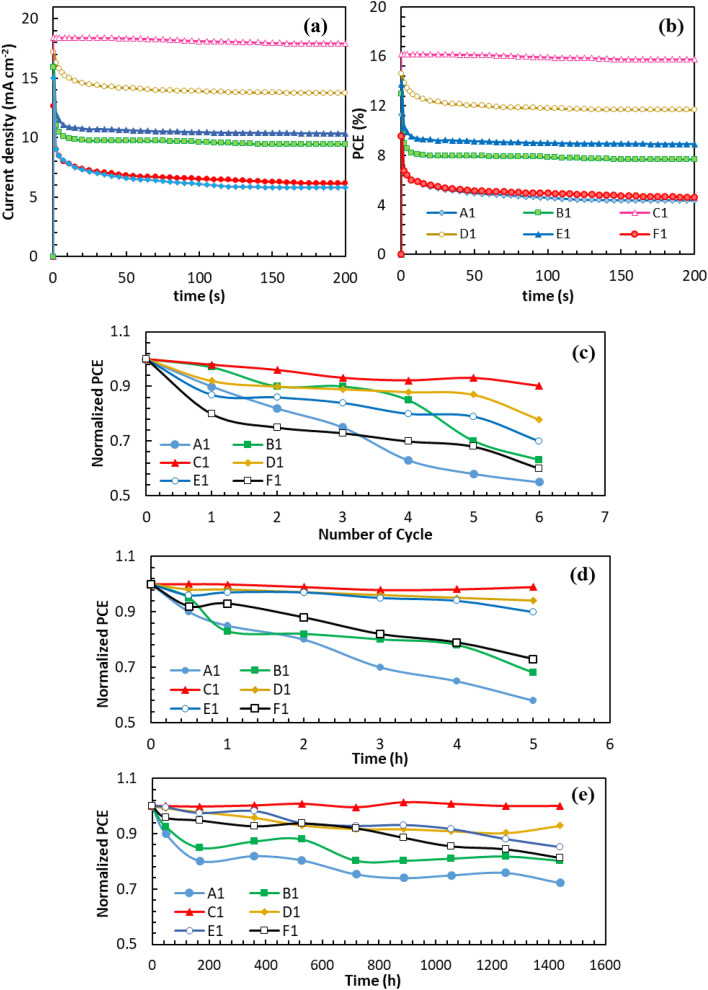
The stabilized (**a**) photocurrent density and (**b**) PCE obtained while holding the PSCs near the maximum power point voltage without pre-exposure under 1 AM 1.5G. Time-dependent normalized PCE of the unencapsulated devices A1, B1, C1, D1, E1 and F1 under (**c**) thermal cycle (one cycle: heating at 85 °C for 30 min and cooling down to room temperature for 1 h in ambient condition with 15 ± 5% RH, (**d**) humidity aging at 90 ± 5% RH and (**e**) humidity aging at 15 ± 5% RH, in the dark. The data were obtained in reverse scan at a scan rate 50 mV s^−1^ under AM 1.5G. Three cells were tested for each kind of device.

To check the reliability of PCE obtained for the fabricated devices, the stabilized photocurrent was measured at voltages corresponding to maximum output power and plotted as a function of time. Figure 4 displays the J−t and PCE-t plots. The comparison of J−t and PCE–t curves for device A1 and C1 indicates that the J_SC_ and PCE of device A1 are unstable during continuous illumination. Such instability property can be improved by making a 2D layer at perovskite/CuSCN interface because of the halide migration inhibition at GBs^45^.

**Figure 5:**
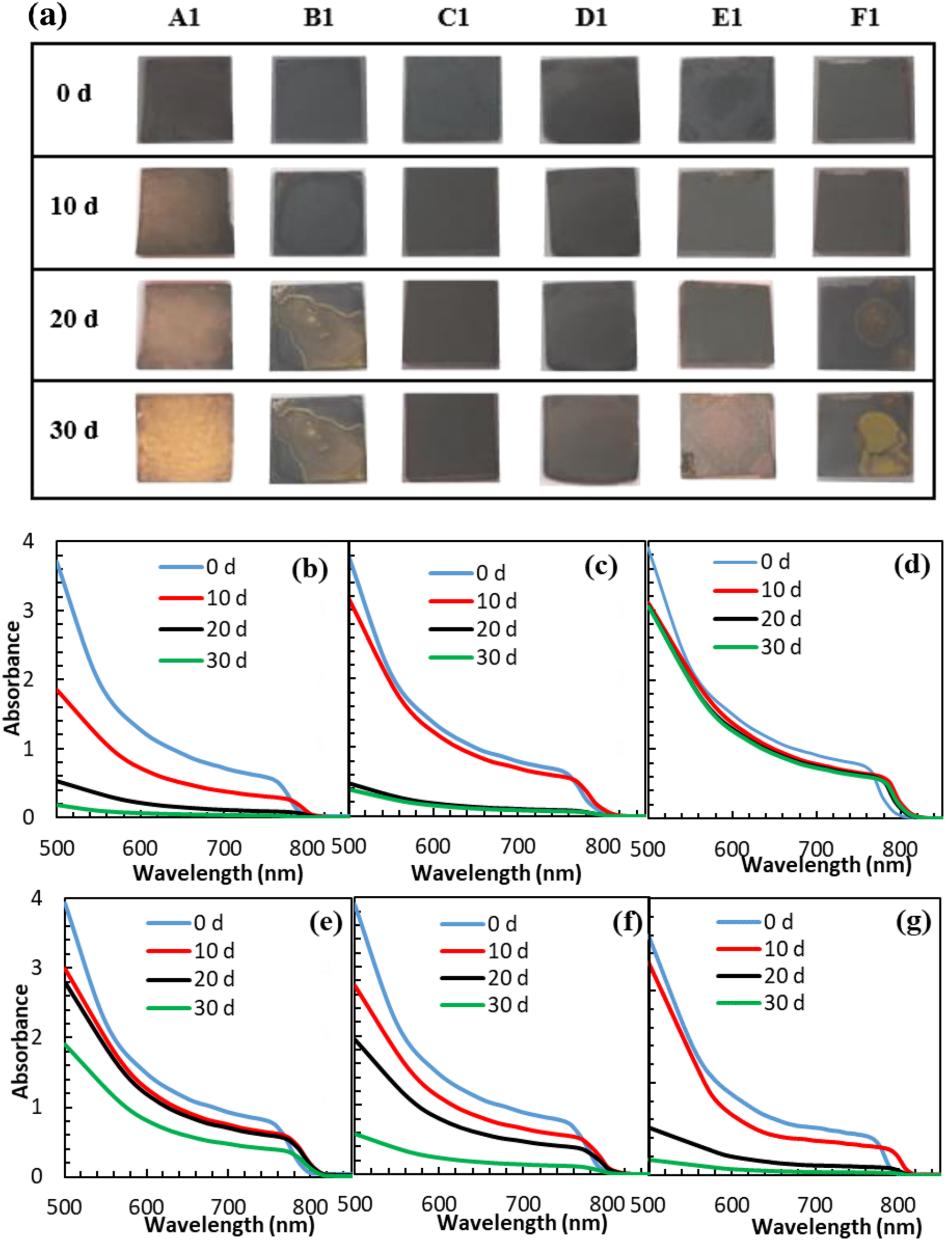
(**a**) Optical images of the perovskite films as a function of humidity exposure time in 90 ± 5% RH. The corresponding UV–Vis absorption spectra of the perovskite films post-treated with (**b**) 0 mg mL^−1^, (**c**) 3 mg mL^−1^, (**d**) 5 mg mL^−1^, (**e**) 7 mg mL^−1^, (**f**) 10 mg mL^−1^ and (**g**) 15 mg mL^−1^ of PDAI and aged in 90 ± 5% RH during the 30 days.

**Figure 6:**
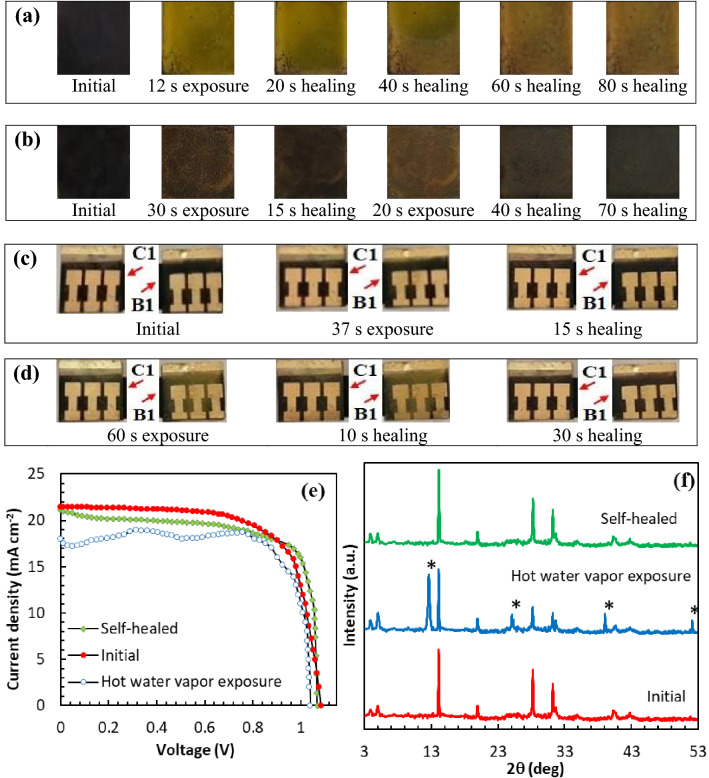
(**a**) Optical images of perovskite films without PDAI post-treatment and (**b**) with 5 mg mL^−1^ PDAI post-treatment, during exposure to hot water vapor and kept in ambient air for self-healing study. (**c**) Optical images of device B1 and C1 showing color changes during the first cycle and (**d**) the second cycle exposure to hot water vapor and kept in ambient air for self-healing study. (**e**) J–V curves of device C1 before and after hot water vapor exposure. (**f**) X-ray diffraction evolution revealing the self-healing process. The symbol ‘*’ represents the peaks of PbI_2_.

The time-dependent degradation of devices is investigated to study the correlation between stability and surface passivation. The thermal stability of unencapsulated devices is studied by keeping them at 85 °C with 15 ± 5% RH (Fig. 4c, and Supplementary Tables S1–S6). The devices A1, B1, C1, D1, E1 and F1 show 54.93%, 62.99%, 90.23%, 79.97%, 69.96% and 60.01% of initial PCE after 6 thermal cycles, respectively. In the case of a pristine device (A1), the PCE is declined during temperature cycles and finally reduced to half of the initial value. In contrast, the PCE of device C1 is obtained 90% after 6 cycles. It is interesting that the declined PCE value of device C1 is gradually recovered again after 3 cycles. The raised temperature in the solar cell speeds up FA^+^ and MA^+^ vibration, and thereby throws out the organic cations from the perovskite lattice, leading to irreversible thermal decomposition^46,47^. Comparably, perovskite films post-treated with the appropriate amount of PDAI exhibit some self-healing properties in the thermal aging test (device C1). In this device, the GBs are passivated by PDAI, and the traps in the heterojunction region of GBs are connected together by diammonium cations. In addition, the ammonium both ends of the molecule can crosslink perovskite grains through hydrogen bonds to form a dense perovskite film and suppress the A^+^ migration far from the [PbX_6_]^4−^ octahedral frame. So, during the self-healing process, the perovskite film will be reconstructed to the 3D lattice structure. Simultaneously, the main problem of perovskite materials is moisture instability because of the hygroscopic nature of 3D perovskite materials. So, 3D perovskite is decomposed into PbI_2_ upon contact with water. The perovskite films (A1 to F1) were kept in home-made equipment with controlled 90 ± 5% RH at room temperature (Supplementary Fig. S8). Figure 5a shows the optical images obtained during the aging test. The UV–Vis spectra of the films are also illustrated in Fig. 5b–g. It is completely obvious that (FAPbI_3_)_0.85_(MAPbBr_3_)_0.15_ is decomposed to the PbI_2_ yellow phase after 30 d. In agreement with the visual observation, the UV–Vis spectra show a regular decline of absorbance intensity. All of the perovskite films passivated by different concentrations of PDAI are degraded at almost the same rate in the first 10 day. Degradation rate is accelerated in the film B1, D1, E1 and F1 after 20 d (especially for film B1 and F1), whereas no further degradation is obtained for the films C1. In order to more deeply investigate the device stability against the moisture, the devices were aged at low and high relative humidity, and the J-V curves were recorded. As shown in Fig. 4d, and Supplementary Tables S7–S12, the PCE of unencapsulated device A1 is sharply decreased and received to 58.02% of initial PCE during 5 h in high 90 ± 5% RH. The PCEs of devices C1 and D1 are maintained 99.01% and 93.99% of the initial value after 5 h, respectively. At higher concentrations of PDAI (15 mg mL^−1^), the excess amount of diammonium salts is trapped in the GBs and deteriorates device stability. Finally, the comparison of device stability in low 15 ± 5% RH shows that the PDAI-treated PSC is less degraded than the (FAPbI_3_)_0.85_(MAPbBr_3_)_0.15_ device (device A1) according to Fig. 4e, and Supplementary Tables S13–S18. The pristine device (A1) shows 72.27% of its initial PCEs under a 15 ± 5% RH. Almost no degradation of device C1 under low 15 ± 5% RH corroborates that the PDAI passivation can effectively hinder moisture destruction. Stability tests confirmed that device C1 is the most stable device. It is due to the post-treatment of PDAI on the perovskite film, which leads to the in-situ growth of the hydrophobic 2D interface layer and thus repels water molecules. This will create a layer of hydrophobic and thus repel water molecules. On the other side, water adsorption on the perovskite surface cannot be completely avoided; the passivation of GBs and defects by PDAI can suppress the perovskite solar cell degradation. The findings strongly propose that PDAI passivation can effectively protect the perovskite film from moisture. So, we thought about it to check the self-healing properties of the perovskite films. Figure 6a and b show a comparison of the color changes for perovskite layer without PDAI post-treatment (film A1) and the perovskite layer with 5 mg mL^−1^ PDAI passivation (film C1) after both were exposed with hot water vapor. In comparison, the film A1 is changed to yellow after 12 s of exposure to water vapor, but the color of the passivated film is remained black during this time and changed to brown after 30 s. So, the pristine film is deteriorated in a humid environment more quickly that perovskite film passivated by PDAI. It is due to the in-situ growth of the 2D (PDAI)PbI_4_ layer on top of the 3D perovskite film, which can create a hydrophobic barrier layer on it. After being kept away from water vapor, the color of the pristine film has remained yellow, and it shows no self-healing behavior after 80 s, but the passivated perovskite film is changed to black color after 15 s of healing. The self-healing perovskite film was immediately exposed to the water vapor again. After 20 s of exposure to water vapor, the film color is changed to brownish-yellow. After being kept away from water vapor for 70 s, the film color is completely changed to black. This wonderful self-healing behavior is also displayed in 3:46 min video (Supplementary Video S1). Whether the perovskite film in the device structure has such a self-healing ability is a little more complicated. In the configured device, the interaction between different layers is also a critical issue, and how much water molecules are absorbed on the ETL and HTL can affect the stability of the perovskite layer. So, the stability of the perovskite layer in the configured solar cell is also investigated. The self-healing ability of two PSCs spin-coating with 3 and 5 mg mL^−1^ PDAI is investigated for a deeper understanding of the PDAI concentration effect. The self-healing capability of devices B1 and C1 is vividly demonstrated in 2:25 min video (Supplementary Video S2). Figure 6c shows a comparison of the color changes for devices B1 and C1. In the first cycle of water vapor contact, the color of device C1 remains almost constant, and a slight color change is observed for device B1 after 37 s of exposure. After 15 s healing step, the device B1 is refreshed again and became completely black. The frequent self-healing ability of devices is studied by device exposure to hot water vapor for 60 s (Fig. 6d). In the second cycle, the color change of device B1 is more obvious than device C1. The adequate amount of PDAI concentration (5 mg mL^−1^) in device C1 has led to the in-situ growth of an ultra-thin and compact 2D (PDAI)_2_PbI_4_ layer on (FAPbI_3_)_0.85_(MAPbBr_3_)_0.15_ film. This hydrophobic 2D layer on the surface of the 3D perovskite film can partially prevent the penetration of water molecules and provides a sufficient amount of moisture stability. The color of device C1 and B1 is returned black after 20 s and 30 s healing step, respectively. The self-healing ability of the device C1 is also confirmed by the J–V curves before and after hot water vapor exposure (Fig. 6e). The J–V curves indicate that the device C1 can heal itself when it is kept away from hot water vapor and the J–V curve can return to its original shape. This self-healing ability is very suitable for the commercialization of perovskite solar cells since once the device is exposed to the humid environment, the solar cell can self-heal to high PCE again in a short time when it returns to sunlight again. Moreover, The self-healing process is studied by XRD analysis (Fig. 6f). The strong peak at 12.7° indicates that PbI_2_ phase is formed during hot water vapor exposure. After self-healing, the PbI_2_ peak is disappeared and the perovskite film is recovered to its original crystal phase.

To further explain such a self-healing ability in the passivated PSCs, it is necessary to explain the exact mechanism of moisture degradation in the perovskite film. The destruction of perovskite films is more considered in the GBs and interfaces^48^. A possible mechanism for irreversible decomposition of perovskite films has been suggested by Choi and coworkers^49^. It is suggested that the irreversible degradation of perovskite materials only happens while both charges and moisture exist simultaneously. In the first step, perovskite films form hydrates in the presence of water molecules. The [PbX_6_]^4−^ octahedra interacts with both organic cations (MA^+^, FA^+^) and H_2_O within the hydrated perovskite^50^. Next, the trapped charge (X^−^) at the defects causes to the organic cation deprotonation and producing volatile molecules like CH_3_CH_2_ (MA) and HC(= NH)NH_2_ (FA)^49^:

2 $$\left(\begin{array}{c}{\mathrm{CH}}_{3}{\mathrm{NH}}_{3}\\ {{\mathrm{HC}(\mathrm{NH}}_{2})}_{2}\end{array}\right){\mathrm{PbX}}_{3}\stackrel{{\mathrm{H}}_{2}\mathrm{O}+\mathrm{TCs}}{\to }{\mathrm{PbX}}_{2}\left(\mathrm{s}\right)+\left(\begin{array}{c}{\mathrm{CH}}_{3}{\mathrm{NH}}_{3}\\ {{\mathrm{HC}(\mathrm{NH}}_{2})}_{2}\end{array}\right)\left(\uparrow \right)+{\mathrm{H}}_{3}{\mathrm{O}}^{+}+{\mathrm{X}}^{-}(\mathrm{aq})$$

3 $$\left(\begin{array}{c}{\mathrm{CH}}_{3}{\mathrm{NH}}_{3}\\ {{\mathrm{HC}(\mathrm{NH}}_{2})}_{2}\end{array}\right){\mathrm{PbX}}_{3}\stackrel{{\mathrm{H}}_{2}\mathrm{O}}{\leftrightarrow }{\mathrm{PbX}}_{2}\left(\mathrm{s}\right)+\left(\begin{array}{c}{\mathrm{CH}}_{3}{\mathrm{NH}}_{3}^{+}\\ {\mathrm{HC}\left({\mathrm{NH}}_{2}\right)}_{2}^{+}\end{array}\right)\left(\mathrm{aq}\right)+{\mathrm{X}}^{-}(\mathrm{aq})$$

where TCs denotes trapped charges, and X indicates halide. The MA and FA evaporation can shift the reactions to the right-hand side, leading to irreversible degradation of perovskite film.

**Figure 7:**
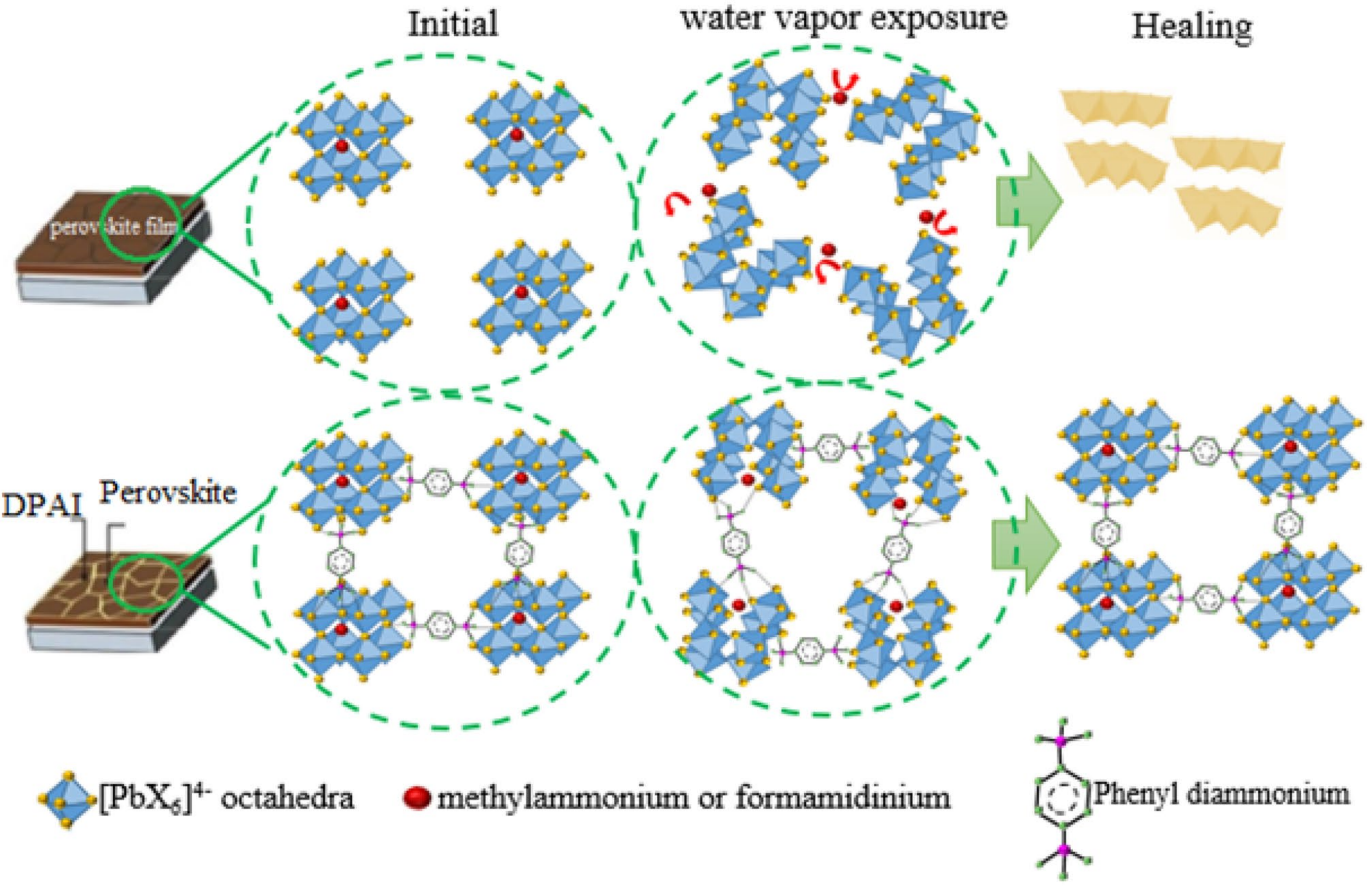
Schematic diagram to show mechanisms for the self-healing properties in device C1.

The self-healing mechanism of the passivated perovskite film is shown in Fig. 7. Interestingly, the hydrogen atoms available in the diammonium cations (PDA) can interact with iodide in [PbX_6_]^4−^ octahedra via hydrogen bonding and create passivation in the GBs of perovskite. The degradation of perovskite is started once the perovskite film is exposed to water vapor. In the pristine film, the perovskite grains start to disintegrate away from each other. So, the MA^+^ and FA^+^ ions are driven through channels and interact with the nearest trapped charges to make volatile molecules. Therefore, the irreversible degradation of pristine perovskite will be happening according to the reaction (2), and the color of the film remains yellow. In the case of passivated perovskite film with PDAI, the GBs are attached strongly because of the presence of two ammonium groups at both ends of the benzene ring. So, the MA^+^ and FA^+^ migrations are partially suppressed through the interconnected GBs. On the other hand, the aromatic ring of phenyl diammonium can suppress the trapped charge migration towards organic cations. All of these can inhibit the deprotonation of organic cations and results in organic cation anchors in the nearest place to the [PbX_6_]^4−^ octahedra rather than escape away. After being kept away from water vapor, the decomposition reaction can take place in the backward direction again, very similar to the two-step synthesis of perovskite film according to the reaction (3). The consecutive decomposition-recombination mechanism expresses the rapid self-healing process in the scaffold perovskite film with the 5 mg mL^−1^ PDAI.

## Methods

### Materials

5 g of formamidinium acetate (Aldrich, 99%) was dissolved in 10 mL hydroiodic acid (57% in water, Merck) and stirred for 2 h in the ice bath for formamidinium iodide (NH_2_CH = NH_2_I = FAI) synthesis. Simultaneously, methylammonium iodide (CH_3_NH_3_Br = MABr) was synthesized by reacting 10 mL methylammonium (33 wt.% in ethanol, Aldrich) and 20 ml hydrobromic acid (48 wt.% in water, Aldrich) in a 100 ml round-bottom flask at 0 °C for 2 h with stirring. The solvent was removed by rotary evaporation at about 50 °C. After washing the precipitate by diethyl ether (99.7%, Merck) for three times and changing the precipitate color to white, the recrystallization process was performed twice with absolute ethanol (99.9%, Merck). *p*-Phenylenediaminium iodide (C_6_H_4_(NH_3_)_2_I_2_ = PDAI) was synthesized by a dropwise addition of 14 mL hydroiodic acid into 5.4 g *p*-Phenylenediamine (PDA 99%, Sigma Aldrich) under stirring at 0 °C for 2 h. The recrystallization process was performed as same as the FAI and MABr synthesis. The participates were dried at 60 °C under a vacuum for 6 h. For CuSCN synthesis as a hole transport material, sodium thiosulfate (Na_2_S_2_O_3_.5H_2_O, Merck) was used as a non-toxic reducing agent. Initially, 50 mL of a 0.1 M aqueous copper (II) sulfate (CuSO_4_.5H_2_O, Merck) solution was mixed with 50 mL of a 0.1 M aqueous Na_2_S_2_O_3_ solution and stirred for 1 h. The solution color change from blue to light green indicated the conversion of Cu (II) ions to Cu (I). Subsequently, 50 mL of a 0.1 M aqueous potassium thiocyanate (KSCN, Merck) solution was slowly added in a dropwise manner for 2 h. After this step, the solution color changed to whitish because of the growth of CuSCN crystals. Finally, the dispersed particle was separated by centrifugation and washed with deionized water and subsequently with absolute ethanol three times. The CuSCN powder was dried in a vacuum oven at 60 °C for 3 h.

### Device fabrication

Fluorine-doped tin-oxide coated glass substrates (FTO, Solaronix, 15 Ω/square, Switzerland) were patterned by etching with zinc powder (98%, Merck) and diluted hydrochloric acid fuming (HCl 37%, Fluka). Then the FTO substrates were cleaned with detergent, acetone (99.8%, Merck), 2-propanol (99.8%, Merck), and distilled water in an ultrasonic bath about 15 min for each step. A compact blocking layer of TiO_2_ (bl-TiO_2_) was deposited on the sintered substrate by spin-coating of a 0.15 M titanium isopropoxide (TTIP 97%, Merck) solution in absolute ethanol at 1500 rpm for 20 s. The film was then sintered at 500 °C for 1 h. The mesoporous TiO_2_ (mp-TiO_2_) layer was deposited by spin-coating TiO_2_ paste (IRASOL PST-20 T) diluted in absolute ethanol by 1:5 weight ratio at 3000 rpm for 20 s, and the substrate was sintered at 500 °C for 30 min. Perovskite solution was prepared by dissolving of FAI, MABr, PbI_2_ (99.99%, IRASOL) and PbBr_2_ (99.99%, IRASOL) with 1 M, 0.2 M, 1.1 M and 0.2 M concentrations in N–N-dimethylformamide (DMF 99.5%, Merck) and dimethyl sulfoxide, (DMSO 99.9%, Merck) mixed solvent (DMF:DMSO, 4:1 volume ratio). For the absorbent layer, 45 μL of prepared perovskite solution was poured onto the mp-TiO_2_ layer and spin-coated at 1000 rpm for 10 s and then 3000 rpm for 40 s. Finally, 500 μL of ethyl acetate (EtOAc 99.5%, Merck) was poured onto the perovskite surface before finishing the spin-coating process. The film was annealed at 130 °C for half one hour. Different concentrations of PDAI were dissolved in 2-propanol (3, 5, 7, 10 and 15 mg mL^−1^) and spin-coated on the surface of the (FAPbI_3_)_0.85_(MAPbBr_3_)_0.15_ films at 3000 rpm for 20 s and the films were annealed at 100 °C for 10 min. Finally, the CuSCN layer was deposited by spin-coating of 50 μL CuSCN solution (40 mg mL^−1^) in dipropyl sulfide (DPS 99.9%, Merck) /acetonitrile (ACN 99.9%, Merck) mixed solvents at 5000 rpm for 20 s. After that, the CuSCN film was annealed at 60 °C for 15 min. Finally, gold thermal evaporation was performed to generate a 50 nm Au metal layer on top of the HTL as a counter electrode. The whole process of the PSCs fabrication was performed in ambient conditions, except for the gold evaporation process.

### Measurement and characterization

The X-ray diffraction (XRD) spectra of the prepared perovskite films were recorded by PANalytical, X’Pert Pro MPD, with an X-ray tube (Cu Kα, λ = 1.5406 Å). The morphology of the films was studied by a field-emission scanning electron microscope (Philips, Model XL30). UV–Vis spectra were observed by the UV–Vis spectrometer (400–1000 nm wavelength range, PerkinElmer Lambda25). Photocurrent density–voltage (J–V) curves were measured by a Keithley 2400 source meter under Am 1.5G (100 mW cm^−2^) simulated light radiation in IRASOL, SIM-1000 system (calibrated by a Thorlabs photodiode) at a scan rate of 50 mV s^−1^. The devices were masked with an aperture area of 0.09 cm^2^ exposed under illumination. Incident photon to current conversion efficiencies (IPCE) was measured by an IRASOL, IPCE-015 equipment. The moisture stability of unencapsulated films or devices was studied by keeping the films or devices in home-made equipment under a relative humidity (RH) of 90 ± 5% at room temperature (27 ± 2). The UV–visible spectra of perovskite films were recorded every 10 d. The thermal stability of unencapsulated devices was studied by aging the devices at 85 °C under RH of 15 ± 5% for 30 min and then cooling down to room temperature for 1 h. J–V curves were obtained in every cycle. The self-healing ability was studied by exposed the unencapsulated devices or films to hot water vapor and kept theme away in the self-healing step.

## Supplementary information

Supplementary Information.

Supplementary Movie S1.

Supplementary Movie S2.

## Data Availability

The datasets generated during the current study are available from the corresponding author on reasonable request.
