# Supplementary material for: p-Phenylenediaminium iodide capping agent enabled self-healing perovskite solar cell
Source: Sci Rep. 2020 Nov 17;10:20011. doi: 10.1038/s41598-020-76365-y (PMC7672076; doi:10.1038/s41598-020-76365-y)
Supplement: Supplementary file 1 — Supplementary Information. [file 41598_2020_76365_MOESM1_ESM.doc]

Supplementary data

*p*-Phenylenediaminium iodide capping agent enabled self-healing perovskite solar cell

Parisa Zardari^1^, Ali Rostami*^1,2^, Hemayat Shekaari^3^

1. Photonic and Nanocrystals Research Laboratory (PNRL), University of Tabriz, Tabriz 5166614761, Iran E-mail: p.zardari@tabriu.ac.ir
2. SP-EPT Laboratory, ASEPE Company, Industrial Park of Advanced Technologies, Tabriz, 5364196795, Iran E-mail: rostami@tabrizu.ac.ir
3. Department of Physical Chemistry, University of Tabriz, Tabriz 5166616471, Iran E-mail: hemayatt@yahoo.com


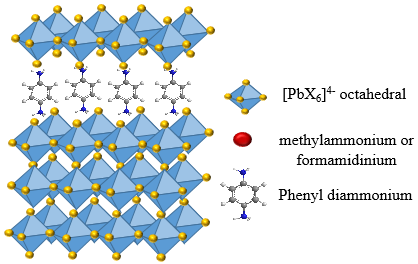


**Figure S1.** Crystal structure of 2D (PDA)PbI_4_ perovskite.


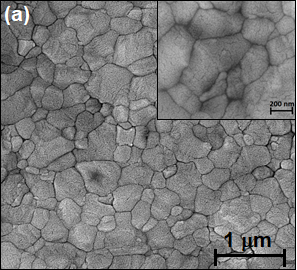

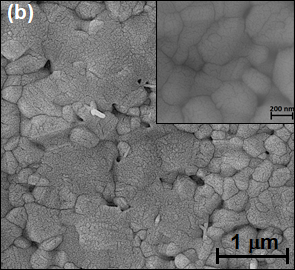

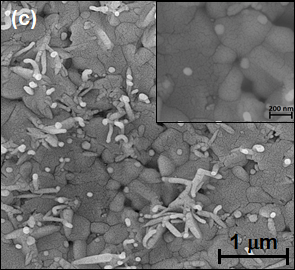

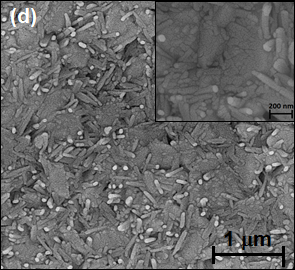


**Figure S2.** Low and high magnification of plane-view SEM images of perovskite films post-treated with (a) 3 mg mL^-1^, (b) 7 mg mL^-1^, (c) 10 mg mL^-1^ and (d) 15 mg mL^-1^ of PDAI. Samples for SEM were prepared by deposition of perovskite films on mp-TiO_2_/bl-TiO_2_/FTO/glass.


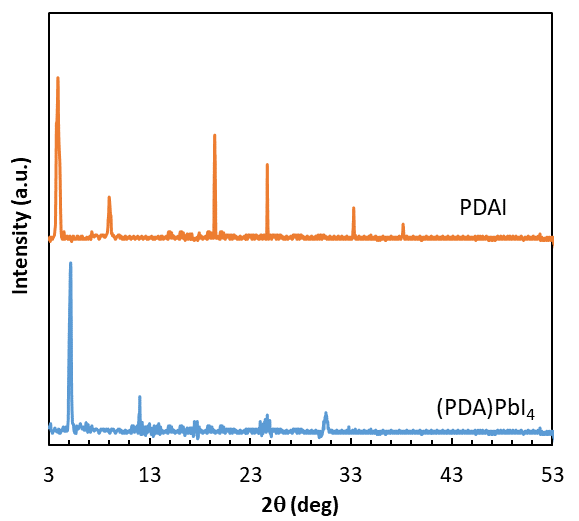


**Figure S3.** XRD patterns of 2D perovskite film and PDAI powder.


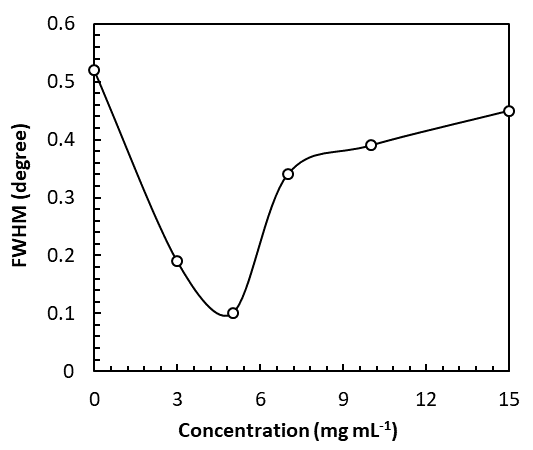


**Figure S4.** The dependence of full width at half maximum (FWHM) for the (110) peak


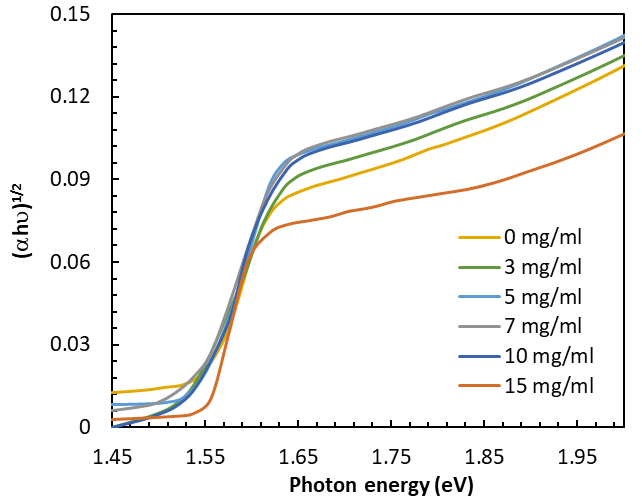


**Figure S5.** (*αhν*)^1/2^ as a function of photon energy for perovskite film post-treated with different concentrations of PDAI deposited on FTO/glass substrate


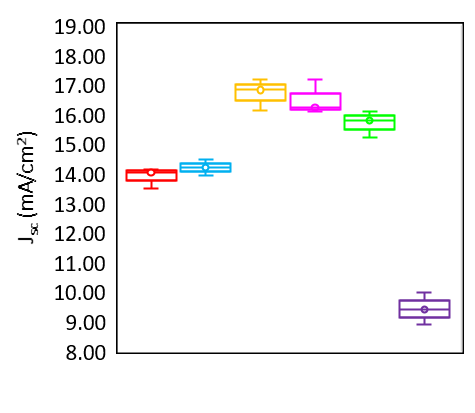


**(a)**

A2 B2 C2 D2 E2 F2


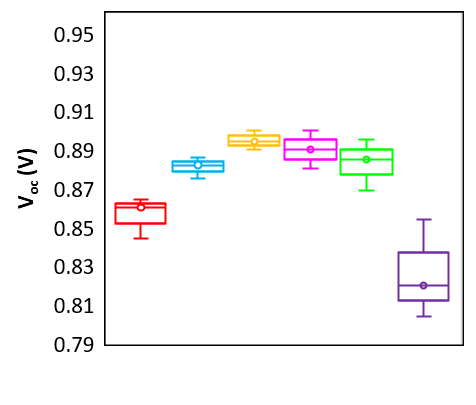


A2 B2 C2 D2 E2 F2

**(b)**


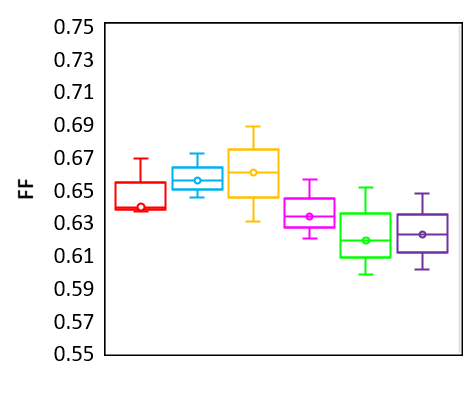


A2 B2 C2 D2 E2 F2

**(c)**


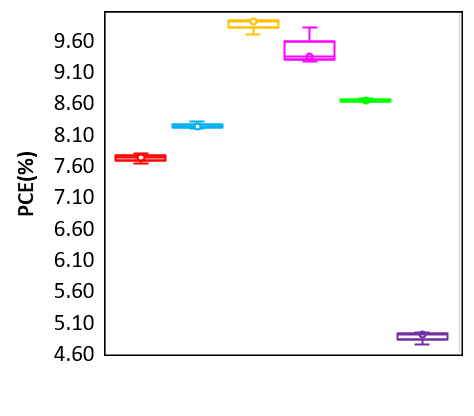


A2 B2 C2 D2 E2 F2

**(d)**


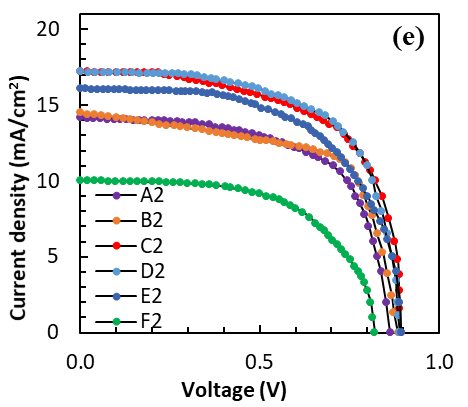

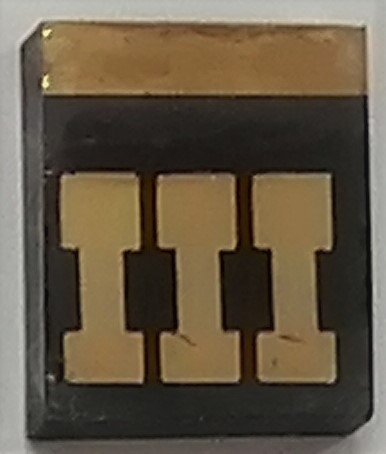

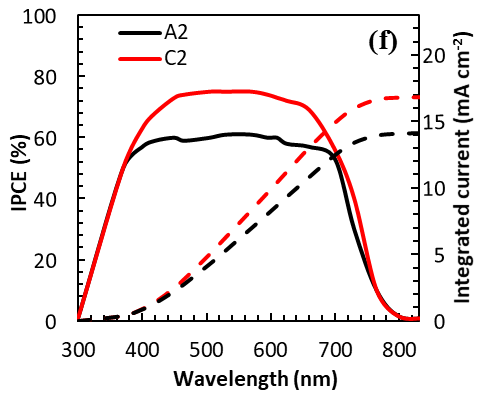


**Figure S6.** Statistic (a) J_SC_, (b) V_OC_, (c) FF, and (d) PCE of devices A2-F2. (e) J–V curves of devices measured in reverse scan at a scan rate of 50 mV s^-1^ under AM 1.5G. (f) The corresponding IPCE spectra of the best performing device C2 and controlled device A2 in which dashed lines stand for current density integrated from IPCE spectra.


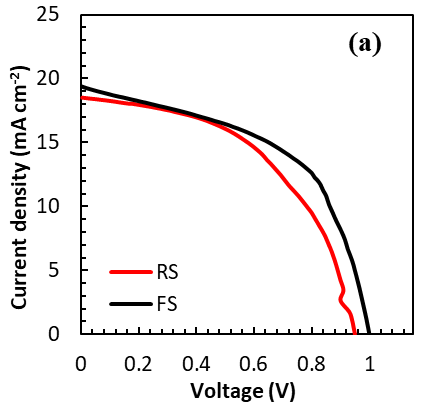

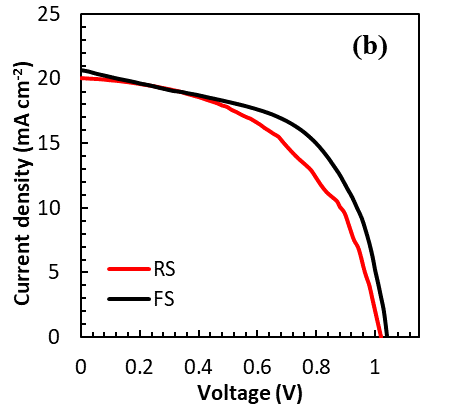

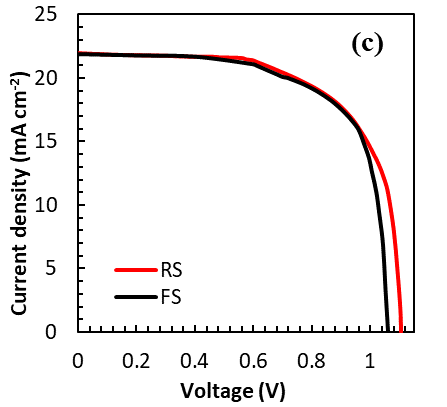

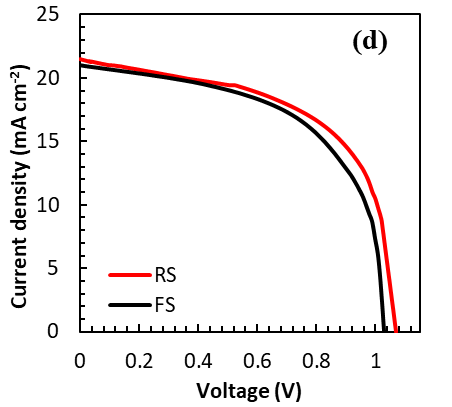

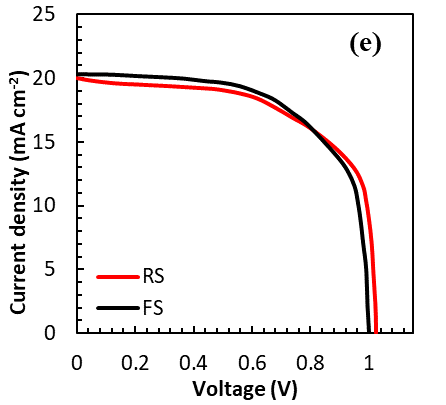

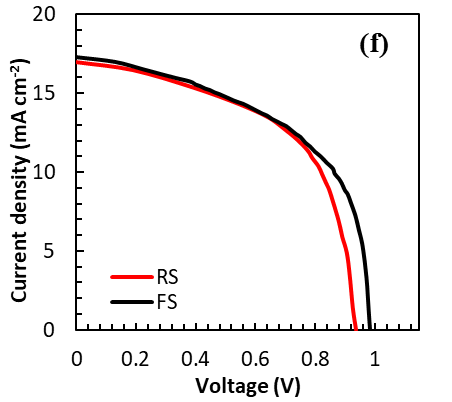


**Figure S7.** J–V curves of (a) device A1, (b) device B1, (c) device C1, (d) device D1, (e) device E1 and (f) device F1 with respect to the reverse scanned (RS) and forward scanned (FS) at a scan rate of 50 mV s^-1^ under AM 1.5G illumination, after one month maintenance in the ambient humidity about ≈15% and dark.


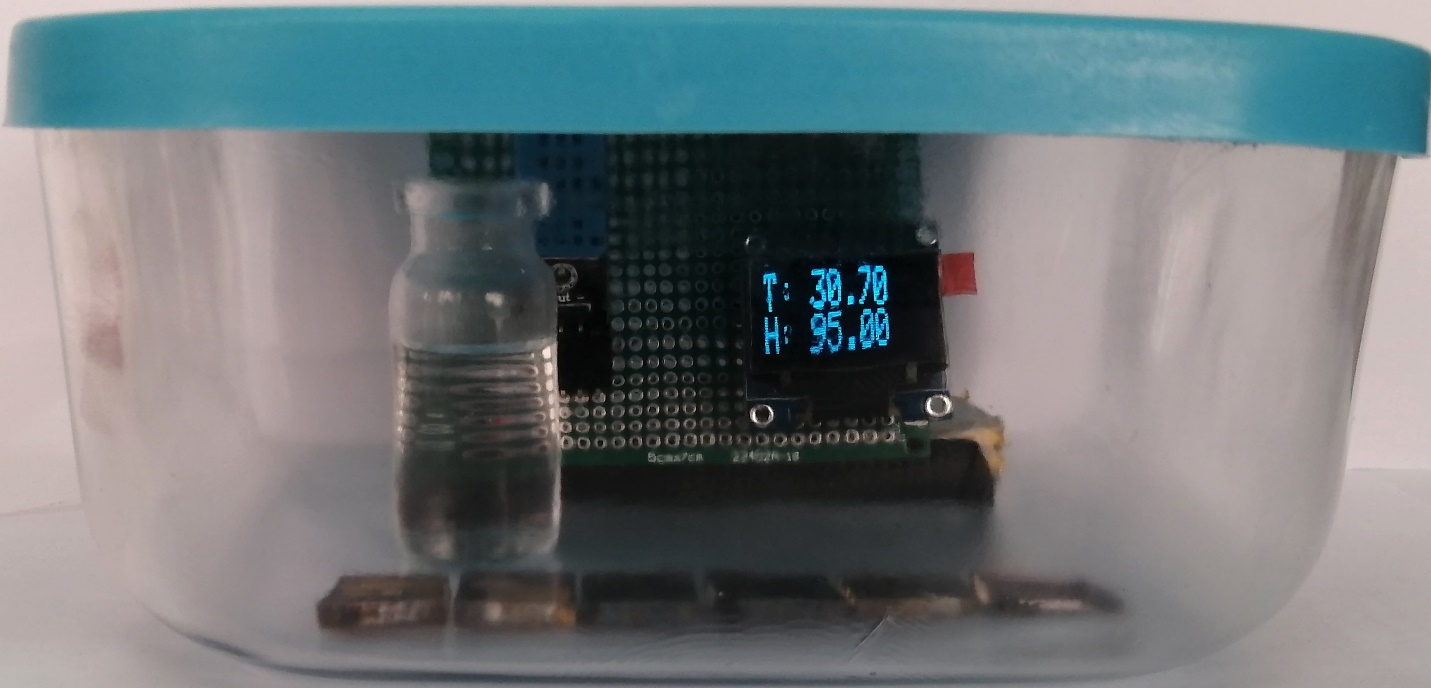


**Figure S8.** Home-made moisture test apparatus equipped with a hygrometer and a bottle with deionized water

**Table S1.** Photovoltaic parameters of the unencapsulated device A1 aged at 85 °C in ambient condition with 15 ± 5% RH, in the dark. The data were recorded in RS at a scan rate 50 mV s^-1^ under AM 1.5G.

| Device | Number of thermal cycle | J_SC_ [mA cm^-2^] | V_OC_ [V] | FF | PCE [%] |
| --- | --- | --- | --- | --- | --- |
| A1 (a) | 0 | 18.78 | 1.02 | 0.64 | 12.20 |
|  | 1 | 17.92 | 0.98 | 0.62 | 10.95 |
|  | 2 | 17.12 | 0.98 | 0.58 | 9.76 |
|  | 3 | 17.01 | 0.95 | 0.55 | 8.82 |
|  | 4 | 16.87 | 0.96 | 0.46 | 7.53 |
|  | 5 | 16.47 | 0.87 | 0.47 | 6.78 |
|  | 6 | 16.21 | 0.87 | 0.45 | 6.36 |
| A1 (b) | 0 | 18.17 | 1.01 | 0.63 | 11.54 |
|  | 1 | 17.76 | 1.00 | 0.59 | 10.52 |
|  | 2 | 17.51 | 0.96 | 0.59 | 9.94 |
|  | 3 | 16.92 | 0.96 | 0.56 | 9.06 |
|  | 4 | 16.74 | 0.95 | 0.47 | 7.55 |
|  | 5 | 16.23 | 0.88 | 0.49 | 7.02 |
|  | 6 | 15.97 | 0.87 | 0.49 | 6.79 |
| A1 (c) | 0 | 18.62 | 1.02 | 0.63 | 11.98 |
|  | 1 | 17.92 | 1.00 | 0.60 | 10.67 |
|  | 2 | 16.75 | 0.98 | 0.58 | 9.59 |
|  | 3 | 16.02 | 0.95 | 0.59 | 8.92 |
|  | 4 | 15.49 | 0.96 | 0.50 | 7.43 |
|  | 5 | 15.03 | 0.92 | 0.50 | 6.94 |
|  | 6 | 14.92 | 0.89 | 0.49 | 6.48 |

**Table S2.** Photovoltaic parameters of the unencapsulated device B1 aged at 85 °C in ambient condition with 15 ± 5% RH, in the dark. The data were recorded in RS at a scan rate 50 mV s^-1^ under AM 1.5G.

| Device | Number of thermal cycle | J_SC_ [mA cm^-2^] | V_OC_ [V] | FF | PCE [%] |
| --- | --- | --- | --- | --- | --- |
| B1 (a) | 0 | 19.92 | 1.04 | 0.61 | 12.62 |
|  | 1 | 19.92 | 1.03 | 0.62 | 12.63 |
|  | 2 | 19.65 | 1.00 | 0.60 | 11.73 |
|  | 3 | 19.62 | 0.97 | 0.61 | 11.52 |
|  | 4 | 19.48 | 0.98 | 0.57 | 10.92 |
|  | 5 | 18.97 | 0.98 | 0.48 | 9.01 |
|  | 6 | 18.85 | 0.97 | 0.44 | 8.06 |
| B1 (b) | 0 | 19.57 | 1.04 | 0.63 | 12.91 |
|  | 1 | 19.32 | 1.04 | 0.60 | 12.12 |
|  | 2 | 19.18 | 1.00 | 0.60 | 11.54 |
|  | 3 | 18.81 | 1.00 | 0.61 | 11.48 |
|  | 4 | 18.82 | 1.00 | 0.58 | 10.88 |
|  | 5 | 18.16 | 0.99 | 0.50 | 8.98 |
|  | 6 | 18.11 | 0.98 | 0.46 | 8.15 |
| B1 (c) | 0 | 19.56 | 1.05 | 0.63 | 12.96 |
|  | 1 | 19.11 | 1.03 | 0.65 | 12.70 |
|  | 2 | 18.79 | 1.00 | 0.61 | 11.45 |
|  | 3 | 18.70 | 1.00 | 0.63 | 11.72 |
|  | 4 | 18.23 | 0.99 | 0.61 | 11.00 |
|  | 5 | 17.61 | 1.00 | 0.51 | 9.02 |
|  | 6 | 17.44 | 1.00 | 0.46 | 8.08 |

**Table S3.** Photovoltaic parameters of the unencapsulated device C1 aged at 85 °C in ambient condition with 15 ± 5% RH, in the dark. The data were recorded in RS at a scan rate 50 mV s^-1^ under AM 1.5G.

| Device | Number of thermal cycle | J_SC_ [mA cm^-2^] | V_OC_ [V] | FF | PCE [%] |
| --- | --- | --- | --- | --- | --- |
| C1 (a) | 0 | 21.32 | 1.11 | 0.68 | 16.05 |
|  | 1 | 21.3 | 1.12 | 0.67 | 15.93 |
|  | 2 | 21.12 | 1.09 | 0.68 | 15.61 |
|  | 3 | 21.08 | 1.01 | 0.70 | 14.94 |
|  | 4 | 21.09 | 1.05 | 0.67 | 14.81 |
|  | 5 | 21.04 | 1.06 | 0.67 | 14.99 |
|  | 6 | 20.98 | 1.02 | 0.68 | 14.51 |
| C1 (b) | 0 | 21.88 | 1.12 | 0.65 | 15.92 |
|  | 1 | 21.69 | 1.10 | 0.65 | 15.49 |
|  | 2 | 21.63 | 1.06 | 0.66 | 15.12 |
|  | 3 | 21.59 | 1.01 | 0.67 | 14.56 |
|  | 4 | 21.63 | 1.07 | 0.63 | 14.58 |
|  | 5 | 21.57 | 1.05 | 0.65 | 14.74 |
|  | 6 | 21.01 | 0.99 | 0.68 | 14.12 |
| C1 (c) | 0 | 21.09 | 1.06 | 0.72 | 16.18 |
|  | 1 | 21.08 | 1.06 | 0.71 | 15.89 |
|  | 2 | 21.05 | 1.05 | 0.71 | 15.72 |
|  | 3 | 21.01 | 1.11 | 0.68 | 15.85 |
|  | 4 | 21.08 | 1.02 | 0.71 | 15.23 |
|  | 5 | 21.01 | 1.04 | 0.70 | 15.25 |
|  | 6 | 20.88 | 1.05 | 0.69 | 15.13 |

**Table S4.** Photovoltaic parameters of the unencapsulated device D1 aged at 85 °C in ambient condition with 15 ± 5% RH, in the dark. The data were recorded in RS at a scan rate 50 mV s^-1^ under AM 1.5G.

| Device | Number of thermal cycle | J_SC_ [mA cm^-2^] | V_OC_ [V] | FF | PCE [%] |
| --- | --- | --- | --- | --- | --- |
| D1 (a) | 0 | 21.56 | 1.03 | 0.67 | 14.88 |
|  | 1 | 21.15 | 0.98 | 0.65 | 13.44 |
|  | 2 | 21.11 | 0.96 | 0.65 | 13.16 |
|  | 3 | 21.13 | 0.96 | 0.64 | 13.01 |
|  | 4 | 21.11 | 0.97 | 0.63 | 12.84 |
|  | 5 | 20.92 | 0.97 | 0.62 | 12.70 |
|  | 6 | 20.48 | 0.98 | 0.57 | 11.41 |
| D1 (b) | 0 | 21.23 | 1.03 | 0.65 | 14.28 |
|  | 1 | 20.99 | 0.98 | 0.64 | 13.23 |
|  | 2 | 20.89 | 0.96 | 0.64 | 12.88 |
|  | 3 | 20.88 | 0.97 | 0.63 | 12.76 |
|  | 4 | 20.86 | 0.95 | 0.64 | 12.62 |
|  | 5 | 20.51 | 0.97 | 0.63 | 12.48 |
|  | 6 | 19.77 | 0.93 | 0.61 | 11.17 |
| D1 (c) | 0 | 20.67 | 1.05 | 0.65 | 14.23 |
|  | 1 | 20.56 | 1.00 | 0.65 | 13.35 |
|  | 2 | 20.32 | 0.98 | 0.66 | 13.14 |
|  | 3 | 20.33 | 0.94 | 0.68 | 12.97 |
|  | 4 | 20.01 | 0.94 | 0.68 | 12.82 |
|  | 5 | 19.97 | 0.96 | 0.66 | 12.68 |
|  | 6 | 19.18 | 0.93 | 0.635743 | 11.34 |

**Table S5.** Photovoltaic parameters of the unencapsulated device E1 aged at 85 °C in ambient condition with 15 ± 5% RH, in the dark. The data were recorded in RS at a scan rate 50 mV s^-1^ under AM 1.5G.

| Device | Number of thermal cycle | J_SC_ [mA cm^-2^] | V_OC_ [V] | FF | PCE [%] |
| --- | --- | --- | --- | --- | --- |
| E1 (a) | 0 | 20.87 | 1.02 | 0.64 | 13.67 |
|  | 1 | 20.01 | 1.00 | 0.60 | 12.01 |
|  | 2 | 19.98 | 0.98 | 0.61 | 11.92 |
|  | 3 | 19.73 | 0.99 | 0.59 | 11.58 |
|  | 4 | 19.72 | 0.97 | 0.58 | 11.06 |
|  | 5 | 19.54 | 1.04 | 0.54 | 10.96 |
|  | 6 | 18.72 | 0.98 | 0.53 | 9.72 |
| E1 (b) | 0 | 20.04 | 1.10 | 0.63 | 13.93 |
|  | 1 | 19.54 | 1.02 | 0.60 | 11.91 |
|  | 2 | 19.53 | 1.01 | 0.60 | 11.82 |
|  | 3 | 19.48 | 1.04 | 0.57 | 11.51 |
|  | 4 | 19.45 | 1.00 | 0.56 | 10.87 |
|  | 5 | 18.91 | 1.02 | 0.56 | 10.82 |
|  | 6 | 17.41 | 1.04 | 0.53 | 9.63 |
| E1 (c) | 0 | 20.12 | 1.10 | 0.63 | 13.92 |
|  | 1 | 19.34 | 1.05 | 0.60 | 12.14 |
|  | 2 | 19.28 | 1.03 | 0.60 | 11.94 |
|  | 3 | 19.16 | 1.02 | 0.60 | 11.74 |
|  | 4 | 18.99 | 0.99 | 0.60 | 11.27 |
|  | 5 | 18.87 | 0.95 | 0.61 | 10.98 |
|  | 6 | 17.13 | 0.93 | 0.61 | 9.68 |

**Table S6.** Photovoltaic parameters of the unencapsulated device F1 aged at 85 °C in ambient condition with 15 ± 5% RH, in the dark. The data were recorded in RS at a scan rate 50 mV s^-1^ under AM 1.5G.

| Device | Number of thermal cycle | J_SC_ [mA cm^-2^] | V_OC_ [V] | FF | PCE [%] |
| --- | --- | --- | --- | --- | --- |
| F1 (a) | 0 | 16.92 | 0.95 | 0.58 | 9.32 |
|  | 1 | 15.05 | 0.92 | 0.56 | 7.73 |
|  | 2 | 14.89 | 0.88 | 0.55 | 7.22 |
|  | 3 | 14.87 | 0.87 | 0.55 | 7.10 |
|  | 4 | 14.75 | 0.84 | 0.54 | 6.73 |
|  | 5 | 14.69 | 0.85 | 0.53 | 6.61 |
|  | 6 | 14.04 | 0.84 | 0.50 | 5.92 |
| F1 (b) | 0 | 16.54 | 0.99 | 0.61 | 9.97 |
|  | 1 | 15.33 | 0.91 | 0.54 | 7.57 |
|  | 2 | 15.28 | 0.87 | 0.54 | 7.18 |
|  | 3 | 14.98 | 0.85 | 0.53 | 6.78 |
|  | 4 | 14.71 | 0.82 | 0.54 | 6.54 |
|  | 5 | 14.57 | 0.84 | 0.55 | 6.74 |
|  | 6 | 13.09 | 0.84 | 0.53 | 5.84 |
| F1 (c) | 0 | 15.82 | 0.99 | 0.61 | 9.51 |
|  | 1 | 14.12 | 0.94 | 0.59 | 7.87 |
|  | 2 | 14.09 | 0.88 | 0.59 | 7.32 |
|  | 3 | 13.86 | 0.90 | 0.58 | 7.23 |
|  | 4 | 13.77 | 0.89 | 0.57 | 7.02 |
|  | 5 | 13.72 | 0.80 | 0.57 | 6.29 |
|  | 6 | 13.19 | 0.76 | 0.58 | 5.81 |

**Table S7.** Photovoltaic parameters of the unencapsulated device A1 exposed to the high relative humidity of 90 ± 5% at room temperature in the dark. The data were recorded in RS at a scan rate 50 mV s^-1^ under AM 1.5G.

| Device | Time [h] | J_SC_ [mA cm^-2^] | V_OC_ [V] | FF | PCE [%] |
| --- | --- | --- | --- | --- | --- |
| A1 (a) | 0 | 18.09 | 1.07 | 0.66 | 12.82 |
|  | 0.5 | 17.21 | 1.07 | 0.65 | 11.92 |
|  | 1 | 16.49 | 1.05 | 0.63 | 10.89 |
|  | 2 | 16.48 | 1.01 | 0.62 | 10.36 |
|  | 3 | 15.34 | 0.98 | 0.58 | 8.75 |
|  | 4 | 15.11 | 0.95 | 0.58 | 8.33 |
|  | 5 | 14.97 | 0.87 | 0.57 | 7.41 |
| A1 (b) | 0 | 18.93 | 1.01 | 0.60 | 11.45 |
|  | 0.5 | 17.28 | 1.03 | 0.58 | 10.35 |
|  | 1 | 16.89 | 1.02 | 0.56 | 9.63 |
|  | 2 | 16.83 | 0.98 | 0.54 | 8.92 |
|  | 3 | 16.13 | 0.94 | 0.54 | 8.21 |
|  | 4 | 15.91 | 0.90 | 0.52 | 7.42 |
|  | 5 | 15.84 | 0.83 | 0.51 | 6.69 |
| A1 (c) | 0 | 18.72 | 1.01 | 0.61 | 11.56 |
|  | 0.5 | 17.52 | 0.96 | 0.59 | 9.96 |
|  | 1 | 16.73 | 0.99 | 0.60 | 9.91 |
|  | 2 | 16.49 | 0.93 | 0.61 | 9.37 |
|  | 3 | 15.98 | 0.88 | 0.58 | 8.12 |
|  | 4 | 15.01 | 0.85 | 0.59 | 7.51 |
|  | 5 | 14.89 | 0.86 | 0.52 | 6.67 |

**Table S8.** Photovoltaic parameters of the unencapsulated device B1 exposed to the high relative humidity of 90 ± 5% at room temperature in the dark. The data were recorded in RS at a scan rate 50 mV s^-1^ under AM 1.5G.

| Device | Time [h] | J_SC_ [mA cm^-2^] | V_OC_ [V] | FF | PCE [%] |
| --- | --- | --- | --- | --- | --- |
| B1 (a) | 0 | 19.87 | 1.04 | 0.61 | 12.56 |
|  | 0.5 | 19.69 | 0.99 | 0.61 | 11.92 |
|  | 1 | 19.01 | 0.91 | 0.61 | 10.56 |
|  | 2 | 18.97 | 0.86 | 0.63 | 10.33 |
|  | 3 | 18.65 | 0.95 | 0.62 | 10.95 |
|  | 4 | 18.39 | 0.91 | 0.62 | 10.36 |
|  | 5 | 17.45 | 0.83 | 0.60 | 8.66 |
| B1 (b) | 0 | 19.13 | 1.02 | 0.63 | 12.24 |
|  | 0.5 | 19.12 | 0.99 | 0.61 | 11.51 |
|  | 1 | 18.42 | 0.89 | 0.61 | 10.02 |
|  | 2 | 18.46 | 0.88 | 0.61 | 9.87 |
|  | 3 | 18.54 | 0.83 | 0.62 | 9.54 |
|  | 4 | 18.84 | 0.81 | 0.61 | 9.31 |
|  | 5 | 17.93 | 0.76 | 0.60 | 8.21 |
| B1 (c) | 0 | 19.39 | 1.06 | 0.63 | 12.98 |
|  | 0.5 | 19.07 | 1.04 | 0.63 | 12.45 |
|  | 1 | 18.41 | 0.93 | 0.63 | 10.76 |
|  | 2 | 18.49 | 0.94 | 0.62 | 10.75 |
|  | 3 | 17.73 | 0.90 | 0.61 | 9.73 |
|  | 4 | 17.91 | 0.88 | 0.62 | 9.80 |
|  | 5 | 18.02 | 0.80 | 0.61 | 8.82 |

**Table S9.** Photovoltaic parameters of the unencapsulated device C1 exposed to the high relative humidity of 90 ± 5% at room temperature in the dark. The data were recorded in RS at a scan rate 50 mV s^-1^ under AM 1.5G.

| Device | Time [h] | J_SC_ [mA cm^-2^] | V_OC_ [V] | FF | PCE [%] |
| --- | --- | --- | --- | --- | --- |
| C1 (a) | 0 | 21.68 | 1.05 | 0.75 | 17.06 |
|  | 0.5 | 21.68 | 1.05 | 0.75 | 17.06 |
|  | 1 | 21.67 | 1.04 | 0.76 | 17.05 |
|  | 2 | 21.67 | 1.04 | 0.75 | 16.88 |
|  | 3 | 21.65 | 1.03 | 0.75 | 16.75 |
|  | 4 | 21.65 | 1.03 | 0.75 | 16.75 |
|  | 5 | 21.65 | 1.04 | 0.75 | 16.88 |
| C1 (b) | 0 | 21.91 | 1.03 | 0.75 | 16.87 |
|  | 0.5 | 21.91 | 1.04 | 0.74 | 16.87 |
|  | 1 | 21.92 | 1.04 | 0.74 | 16.84 |
|  | 2 | 21.91 | 1.01 | 0.75 | 16.52 |
|  | 3 | 21.91 | 0.99 | 0.75 | 16.22 |
|  | 4 | 21.91 | 0.99 | 0.76 | 16.44 |
|  | 5 | 21.89 | 1.02 | 0.75 | 16.72 |
| C1 (c) | 0 | 21.86 | 1.10 | 0.73 | 17.52 |
|  | 0.5 | 21.85 | 1.10 | 0.73 | 17.52 |
|  | 1 | 21.86 | 1.08 | 0.74 | 17.51 |
|  | 2 | 21.86 | 1.08 | 0.74 | 17.54 |
|  | 3 | 21.84 | 1.08 | 0.74 | 17.47 |
|  | 4 | 21.85 | 1.07 | 0.74 | 17.33 |
|  | 5 | 21.85 | 1.06 | 0.75 | 17.36 |

**Table S10.** Photovoltaic parameters of the unencapsulated device D1 exposed to the high relative humidity of 90 ± 5% at room temperature in the dark. The data were recorded in RS at a scan rate 50 mV s^-1^ under AM 1.5G.

| Device | Time [h] | J_SC_ [mA cm^-2^] | V_OC_ [V] | FF | PCE [%] |
| --- | --- | --- | --- | --- | --- |
| D1 (a) | 0 | 21.72 | 1.03 | 0.68 | 15.22 |
|  | 0.5 | 21.72 | 1.03 | 0.68 | 15.21 |
|  | 1 | 21.73 | 1.03 | 0.68 | 15.29 |
|  | 2 | 21.72 | 1.00 | 0.69 | 14.99 |
|  | 3 | 21.71 | 0.98 | 0.69 | 14.73 |
|  | 4 | 21.71 | 0.98 | 0.68 | 14.47 |
|  | 5 | 21.68 | 0.96 | 0.69 | 14.39 |
| D1 (b) | 0 | 21.54 | 1.03 | 0.68 | 15.08 |
|  | 0.5 | 21.48 | 1.00 | 0.68 | 14.60 |
|  | 1 | 21.48 | 1.02 | 0.67 | 14.75 |
|  | 2 | 21.48 | 1.00 | 0.68 | 14.64 |
|  | 3 | 21.47 | 0.99 | 0.68 | 14.48 |
|  | 4 | 21.45 | 0.97 | 0.69 | 14.33 |
|  | 5 | 21.32 | 0.95 | 0.70 | 14.21 |
| D1 (c) | 0 | 21.68 | 1.05 | 0.67 | 15.18 |
|  | 0.5 | 21.63 | 1.05 | 0.65 | 14.78 |
|  | 1 | 21.65 | 1.02 | 0.66 | 14.53 |
|  | 2 | 21.68 | 1.01 | 0.66 | 14.51 |
|  | 3 | 21.65 | 1.03 | 0.65 | 14.43 |
|  | 4 | 21.61 | 1.04 | 0.64 | 14.40 |
|  | 5 | 21.59 | 1.04 | 0.63 | 14.14 |

**Table S11.** Photovoltaic parameters of the unencapsulated device E1 exposed to the high relative humidity of 90 ± 5% at room temperature in the dark. The data were recorded in RS at a scan rate 50 mV s^-1^ under AM 1.5G.

| Device | Time [h] | J_SC_ [mA cm^-2^] | V_OC_ [V] | FF | PCE [%] |
| --- | --- | --- | --- | --- | --- |
| E1 (a) | 0 | 20.08 | 1.02 | 0.66 | 13.55 |
|  | 0.5 | 19.19 | 1.04 | 0.65 | 13.01 |
|  | 1 | 19.57 | 1.03 | 0.65 | 13.14 |
|  | 2 | 19.57 | 1.05 | 0.64 | 13.12 |
|  | 3 | 19.43 | 1.00 | 0.66 | 12.87 |
|  | 4 | 19.38 | 1.01 | 0.65 | 12.68 |
|  | 5 | 19.00 | 1.02 | 0.63 | 12.20 |
| E1 (b) | 0 | 20.02 | 1.02 | 0.64 | 13.12 |
|  | 0.5 | 19.54 | 1.00 | 0.64 | 12.56 |
|  | 1 | 19.63 | 1.03 | 0.63 | 12.73 |
|  | 2 | 19.61 | 1.03 | 0.63 | 12.71 |
|  | 3 | 19.55 | 1.03 | 0.62 | 12.46 |
|  | 4 | 19.12 | 1.04 | 0.62 | 12.36 |
|  | 5 | 18.99 | 1.02 | 0.62 | 11.99 |
| E1 (c) | 0 | 20.12 | 1.02 | 0.66 | 13.51 |
|  | 0.5 | 19.88 | 1.02 | 0.64 | 12.98 |
|  | 1 | 19.91 | 1.03 | 0.64 | 13.10 |
|  | 2 | 19.92 | 1.03 | 0.64 | 13.14 |
|  | 3 | 19.45 | 1.05 | 0.63 | 12.82 |
|  | 4 | 19.21 | 1.04 | 0.64 | 12.73 |
|  | 5 | 18.99 | 1.00 | 0.63 | 11.96 |

**Table S12.** Photovoltaic parameters of the unencapsulated device F1 exposed to the high relative humidity of 90 ± 5% at room temperature in the dark. The data were recorded in RS at a scan rate 50 mV s^-1^ under AM 1.5G.

| Device | Time [h] | J_SC_ [mA cm^-2^] | V_OC_ [V] | FF | PCE [%] |
| --- | --- | --- | --- | --- | --- |
| F1 (a) | 0 | 15.95 | 0.93 | 0.60 | 8.86 |
|  | 0.5 | 15.12 | 0.94 | 0.60 | 8.51 |
|  | 1 | 15.23 | 0.95 | 0.59 | 8.51 |
|  | 2 | 15.24 | 0.96 | 0.59 | 8.59 |
|  | 3 | 15.24 | 0.92 | 0.58 | 8.13 |
|  | 4 | 14.96 | 0.94 | 0.57 | 8.03 |
|  | 5 | 14.96 | 0.94 | 0.57 | 7.97 |
| F1 (b) | 0 | 15.88 | 0.94 | 0.59 | 8.83 |
|  | 0.5 | 15.12 | 0.93 | 0.60 | 8.48 |
|  | 1 | 15.12 | 0.94 | 0.59 | 8.37 |
|  | 2 | 15.10 | 0.97 | 0.57 | 8.38 |
|  | 3 | 14.98 | 1.00 | 0.56 | 8.39 |
|  | 4 | 14.35 | 1.01 | 0.57 | 8.28 |
|  | 5 | 14.39 | 0.99 | 0.56 | 7.95 |
| F1 (c) | 0 | 16.01 | 0.92 | 0.61 | 9.02 |
|  | 0.5 | 15.23 | 0.93 | 0.61 | 8.63 |
|  | 1 | 15.45 | 0.95 | 0.61 | 8.99 |
|  | 2 | 15.45 | 0.96 | 0.60 | 8.91 |
|  | 3 | 14.78 | 1.02 | 0.59 | 8.87 |
|  | 4 | 14.69 | 1.00 | 0.60 | 8.79 |
|  | 5 | 14.45 | 0.94 | 0.60 | 8.11 |

**Table S13.** Photovoltaic parameters of the unencapsulated device A1 exposed to the low relative humidity of 15 ± 5% at room temperature in the dark. The data were recorded in RS at a scan rate 50 mV s^-1^ under AM 1.5G.

| Device | Time [h] | J_SC_ [mA cm^-2^] | V_OC_ [V] | FF | PCE [%] |
| --- | --- | --- | --- | --- | --- |
| A1 (a) | 0 | 18.75 | 1.00 | 0.60 | 11.25 |
|  | 48 | 18.67 | 0.92 | 0.59 | 10.11 |
|  | 168 | 18.63 | 0.82 | 0.59 | 9.00 |
|  | 360 | 18.61 | 0.85 | 0.58 | 9.21 |
|  | 528 | 18.59 | 0.84 | 0.58 | 9.03 |
|  | 720 | 18.59 | 0.95 | 0.48 | 8.48 |
|  | 888 | 18.02 | 0.96 | 0.48 | 8.33 |
|  | 1056 | 17.41 | 0.99 | 0.49 | 8.46 |
|  | 1248 | 17.39 | 1.00 | 0.49 | 8.52 |
|  | 1440 | 17.22 | 1.00 | 0.47 | 8.13 |
| A1 (b) | 0 | 18.48 | 1.00 | 0.60 | 11.12 |
|  | 48 | 18.43 | 0.95 | 0.58 | 10.11 |
|  | 168 | 18.38 | 0.87 | 0.56 | 8.93 |
|  | 360 | 18.22 | 0.93 | 0.54 | 9.13 |
|  | 528 | 18.24 | 0.91 | 0.54 | 8.97 |
|  | 720 | 18.29 | 0.98 | 0.47 | 8.38 |
|  | 888 | 17.99 | 0.89 | 0.51 | 8.21 |
|  | 1056 | 17.83 | 0.92 | 0.51 | 8.35 |
|  | 1248 | 17.23 | 0.96 | 0.51 | 8.46 |
|  | 1440 | 16.79 | 0.96 | 0.50 | 8.04 |
| A1 (c) | 0 | 19.09 | 0.98 | 0.61 | 11.38 |
|  | 48 | 19.09 | 0.90 | 0.59 | 10.14 |
|  | 168 | 19.06 | 0.80 | 0.60 | 9.12 |
|  | 360 | 19.06 | 0.80 | 0.61 | 9.32 |
|  | 528 | 19.01 | 0.83 | 0.58 | 9.15 |
|  | 720 | 19.00 | 0.96 | 0.47 | 8.58 |
|  | 888 | 18.21 | 0.99 | 0.47 | 8.45 |
|  | 1056 | 18.06 | 1.00 | 0.47 | 8.52 |
|  | 1248 | 17.97 | 1.00 | 0.48 | 8.63 |
|  | 1440 | 16.56 | 0.99 | 0.50 | 8.22 |

**Table S14.** Photovoltaic parameters of the unencapsulated device B1 exposed to the low relative humidity of 15 ± 5% at room temperature in the dark. The data were recorded in RS at a scan rate 50 mV s^-1^ under AM 1.5G.

| Device | Time [h] | J_SC_ [mA cm^-2^] | V_OC_ [V] | FF | PCE [%] |
| --- | --- | --- | --- | --- | --- |
| B1 (a) | 0 | 19.894 | 1.05 | 0.62 | 12.96 |
|  | 48 | 19.87 | 1.05 | 0.58 | 12.05 |
|  | 168 | 19.85 | 1.05 | 0.53 | 11.00 |
|  | 360 | 20.04 | 1.06 | 0.53 | 11.30 |
|  | 528 | 20.25 | 1.09 | 0.52 | 11.43 |
|  | 720 | 20.23 | 1.03 | 0.50 | 10.40 |
|  | 888 | 19.89 | 1.02 | 0.51 | 10.39 |
|  | 1056 | 19.02 | 1.08 | 0.51 | 10.50 |
|  | 1248 | 19.02 | 1.07 | 0.52 | 10.62 |
|  | 1440 | 19.00 | 1.07 | 0.51 | 10.40 |
| B1 (b) | 0 | 19.28 | 1.05 | 0.64 | 12.92 |
|  | 48 | 19.28 | 1.01 | 0.61 | 11.93 |
|  | 168 | 19.27 | 1.00 | 0.57 | 10.97 |
|  | 360 | 20.01 | 1.01 | 0.56 | 11.29 |
|  | 528 | 20.02 | 1.03 | 0.55 | 11.36 |
|  | 720 | 19.62 | 1.02 | 0.52 | 10.37 |
|  | 888 | 19.73 | 1.01 | 0.52 | 10.36 |
|  | 1056 | 19.29 | 1.06 | 0.51 | 10.47 |
|  | 1248 | 18.98 | 1.07 | 0.52 | 10.55 |
|  | 1440 | 18.95 | 1.03 | 0.53 | 10.35 |
| B1 (c) | 0 | 19.75 | 1.06 | 0.62 | 12.99 |
|  | 48 | 19.69 | 1.00 | 0.61 | 12.03 |
|  | 168 | 19.68 | 0.98 | 0.57 | 11.03 |
|  | 360 | 20.11 | 0.99 | 0.57 | 11.31 |
|  | 528 | 20.25 | 1.01 | 0.56 | 11.40 |
|  | 720 | 20.21 | 1.03 | 0.50 | 10.42 |
|  | 888 | 20.01 | 1.00 | 0.52 | 10.44 |
|  | 1056 | 19.79 | 1.02 | 0.52 | 10.52 |
|  | 1248 | 19.71 | 1.06 | 0.51 | 10.62 |
|  | 1440 | 19.19 | 1.05 | 0.52 | 10.44 |

**Table S15.** Photovoltaic parameters of the unencapsulated device C1 exposed to the low relative humidity of 15 ± 5% at room temperature in the dark. The data were recorded in RS at a scan rate 50 mV s^-1^ under AM 1.5G.

| Device | Time [h] | J_SC_ [mA cm^-2^] | V_OC_ [V] | FF | PCE [%] |
| --- | --- | --- | --- | --- | --- |
| C1 (a) | 0 | 21.58 | 1.09 | 0.69 | 16.28 |
|  | 48 | 21.56 | 1.09 | 0.69 | 16.25 |
|  | 168 | 21.83 | 1.08 | 0.69 | 16.22 |
|  | 360 | 21.83 | 1.09 | 0.68 | 16.25 |
|  | 528 | 21.88 | 1.10 | 0.68 | 16.30 |
|  | 720 | 21.88 | 1.10 | 0.67 | 16.14 |
|  | 888 | 21.85 | 1.12 | 0.67 | 16.39 |
|  | 1056 | 21.61 | 1.14 | 0.66 | 16.31 |
|  | 1248 | 21.58 | 1.13 | 0.67 | 16.27 |
|  | 1440 | 21.38 | 1.13 | 0.67 | 16.25 |
| C1 (b) | 0 | 21.64 | 1.07 | 0.69 | 15.93 |
|  | 48 | 21.33 | 1.01 | 0.74 | 15.94 |
|  | 168 | 21.51 | 0.99 | 0.74 | 15.82 |
|  | 360 | 21.49 | 0.99 | 0.75 | 15.96 |
|  | 528 | 21.98 | 0.97 | 0.75 | 16.06 |
|  | 720 | 21.97 | 1.09 | 0.66 | 15.80 |
|  | 888 | 21.92 | 1.12 | 0.66 | 16.16 |
|  | 1056 | 21.98 | 1.12 | 0.65 | 16.06 |
|  | 1248 | 21.94 | 1.11 | 0.65 | 15.83 |
|  | 1440 | 21.97 | 1.09 | 0.66 | 15.85 |
| C1 (c) | 0 | 21.96 | 1.05 | 0.70 | 16.10 |
|  | 48 | 21.81 | 1.08 | 0.68 | 16.09 |
|  | 168 | 21.99 | 1.12 | 0.65 | 16.04 |
|  | 360 | 22.01 | 1.11 | 0.66 | 16.10 |
|  | 528 | 22.10 | 1.13 | 0.65 | 16.23 |
|  | 720 | 22.17 | 1.11 | 0.65 | 16.02 |
|  | 888 | 22.15 | 1.14 | 0.65 | 16.35 |
|  | 1056 | 22.01 | 1.13 | 0.65 | 16.23 |
|  | 1248 | 22.53 | 1.12 | 0.64 | 16.09 |
|  | 1440 | 22.42 | 1.12 | 0.64 | 16.10 |

**Table S16.** Photovoltaic parameters of the unencapsulated device D1 exposed to the low relative humidity of 15 ± 5% at room temperature in the dark. The data were recorded in RS at a scan rate 50 mV s^-1^ under AM 1.5G.

| Device | Time [h] | J_SC_ [mA cm^-2^] | V_OC_ [V] | FF | PCE [%] |
| --- | --- | --- | --- | --- | --- |
| D1 (a) | 0 | 21.28 | 1.06 | 0.65 | 14.69 |
|  | 48 | 21.22 | 1.05 | 0.65 | 14.55 |
|  | 168 | 21.29 | 1.04 | 0.65 | 14.36 |
|  | 360 | 21.31 | 1.05 | 0.63 | 14.05 |
|  | 528 | 21.30 | 1.07 | 0.60 | 13.69 |
|  | 720 | 21.31 | 1.07 | 0.59 | 13.47 |
|  | 888 | 21.31 | 1.07 | 0.59 | 13.46 |
|  | 1056 | 21.27 | 1.06 | 0.59 | 13.34 |
|  | 1248 | 21.19 | 1.08 | 0.58 | 13.26 |
|  | 1440 | 21.22 | 1.11 | 0.58 | 13.65 |
| D1 (b) | 0 | 21.19 | 1.07 | 0.64 | 14.56 |
|  | 48 | 21.18 | 1.08 | 0.63 | 14.46 |
|  | 168 | 21.15 | 1.07 | 0.63 | 14.27 |
|  | 360 | 21.39 | 1.05 | 0.62 | 13.96 |
|  | 528 | 21.41 | 1.05 | 0.60 | 13.52 |
|  | 720 | 21.56 | 1.05 | 0.59 | 13.35 |
|  | 888 | 21.55 | 1.05 | 0.59 | 13.37 |
|  | 1056 | 21.57 | 1.04 | 0.59 | 13.25 |
|  | 1248 | 21.16 | 1.07 | 0.58 | 13.14 |
|  | 1440 | 21.19 | 1.10 | 0.58 | 13.54 |
| D1 (c) | 0 | 20.87 | 1.03 | 0.68 | 14.61 |
|  | 48 | 20.59 | 1.07 | 0.66 | 14.49 |
|  | 168 | 20.33 | 1.05 | 0.67 | 14.27 |
|  | 360 | 20.96 | 1.01 | 0.66 | 13.99 |
|  | 528 | 21.07 | 1.09 | 0.59 | 13.59 |
|  | 720 | 21.47 | 1.10 | 0.57 | 13.40 |
|  | 888 | 21.49 | 1.09 | 0.57 | 13.37 |
|  | 1056 | 21.31 | 1.08 | 0.58 | 13.31 |
|  | 1248 | 20.91 | 1.07 | 0.59 | 13.19 |
|  | 1440 | 20.86 | 1.08 | 0.60 | 13.55 |

**Table S17.** Photovoltaic parameters of the unencapsulated device E1 exposed to the low relative humidity of 15 ± 5% at room temperature in the dark. The data were recorded in RS at a scan rate 50 mV s^-1^ under AM 1.5G.

| Device | Time [h] | J_SC_ [mA cm^-2^] | V_OC_ [V] | FF | PCE [%] |
| --- | --- | --- | --- | --- | --- |
| E1 (a) | 0 | 18.96 | 1.09 | 0.67 | 13.86 |
|  | 48 | 19.28 | 1.09 | 0.66 | 13.81 |
|  | 168 | 19.65 | 1.04 | 0.66 | 13.50 |
|  | 360 | 19.66 | 1.07 | 0.65 | 13.61 |
|  | 528 | 19.82 | 1.02 | 0.64 | 13.00 |
|  | 720 | 19.97 | 1.02 | 0.63 | 12.86 |
|  | 888 | 19.31 | 1.06 | 0.63 | 12.91 |
|  | 1056 | 19.12 | 1.07 | 0.62 | 12.70 |
|  | 1248 | 18.56 | 1.10 | 0.60 | 12.21 |
|  | 1440 | 18.29 | 1.10 | 0.59 | 11.82 |
| E1 (b) | 0 | 19.32 | 1.10 | 0.65 | 13.79 |
|  | 48 | 19.56 | 1.10 | 0.64 | 13.76 |
|  | 168 | 19.97 | 1.07 | 0.63 | 13.44 |
|  | 360 | 19.96 | 1.08 | 0.63 | 13.52 |
|  | 528 | 19.97 | 1.05 | 0.62 | 12.94 |
|  | 720 | 19.98 | 1.03 | 0.62 | 12.79 |
|  | 888 | 19.76 | 1.05 | 0.62 | 12.84 |
|  | 1056 | 19.34 | 1.09 | 0.60 | 12.66 |
|  | 1248 | 18.45 | 1.12 | 0.59 | 12.17 |
|  | 1440 | 18.00 | 1.11 | 0.59 | 11.74 |
| E1 (c) | 0 | 19.93 | 1.06 | 0.66 | 13.90 |
|  | 48 | 20.01 | 1.08 | 0.64 | 13.83 |
|  | 168 | 20.05 | 1.06 | 0.64 | 13.54 |
|  | 360 | 20.08 | 1.06 | 0.64 | 13.65 |
|  | 528 | 20.09 | 1.03 | 0.63 | 13.05 |
|  | 720 | 20.10 | 1.00 | 0.64 | 12.90 |
|  | 888 | 19.65 | 1.05 | 0.63 | 12.98 |
|  | 1056 | 19.01 | 1.06 | 0.63 | 12.75 |
|  | 1248 | 18.89 | 1.03 | 0.63 | 12.22 |
|  | 1440 | 17.93 | 1.07 | 0.62 | 11.84 |

**Table S18.** Photovoltaic parameters of the unencapsulated device F1 exposed to the low relative humidity of 15 ± 5% at room temperature in the dark. The data were recorded in RS at a scan rate 50 mV s^-1^ under AM 1.5G.

| Device | Time [h] | J_SC_ [mA cm^-2^] | V_OC_ [V] | FF | PCE [%] |
| --- | --- | --- | --- | --- | --- |
| F1 (a) | 0 | 16.803 | 0.97 | 0.60 | 9.82 |
|  | 48 | 16.78 | 0.93 | 0.60 | 9.39 |
|  | 168 | 16.99 | 0.93 | 0.59 | 9.30 |
|  | 360 | 17.02 | 0.90 | 0.59 | 9.08 |
|  | 528 | 17.06 | 0.93 | 0.58 | 9.20 |
|  | 720 | 17.19 | 0.92 | 0.57 | 9.05 |
|  | 888 | 16.03 | 0.95 | 0.57 | 8.70 |
|  | 1056 | 15.12 | 0.98 | 0.57 | 8.41 |
|  | 1248 | 14.90 | 0.97 | 0.57 | 8.27 |
|  | 1440 | 14.65 | 0.98 | 0.56 | 8.03 |
| F1 (b) | 0 | 16.10 | 0.93 | 0.64 | 9.63 |
|  | 48 | 16.02 | 0.96 | 0.60 | 9.25 |
|  | 168 | 16.19 | 0.96 | 0.59 | 9.12 |
|  | 360 | 16.35 | 0.96 | 0.57 | 8.95 |
|  | 528 | 16.78 | 0.96 | 0.56 | 9.05 |
|  | 720 | 16.94 | 0.92 | 0.57 | 8.84 |
|  | 888 | 16.19 | 0.94 | 0.56 | 8.53 |
|  | 1056 | 15.45 | 0.95 | 0.56 | 8.23 |
|  | 1248 | 14.81 | 1.00 | 0.55 | 8.17 |
|  | 1440 | 14.02 | 1.02 | 0.55 | 7.83 |
| F1 (c) | 0 | 16.20 | 0.95 | 0.63 | 9.68 |
|  | 48 | 16.70 | 0.91 | 0.61 | 9.27 |
|  | 168 | 16.68 | 0.90 | 0.61 | 9.16 |
|  | 360 | 16.89 | 0.93 | 0.57 | 8.97 |
|  | 528 | 16.88 | 0.97 | 0.55 | 9.05 |
|  | 720 | 16.88 | 0.98 | 0.54 | 8.90 |
|  | 888 | 15.45 | 1.03 | 0.54 | 8.57 |
|  | 1056 | 14.92 | 1.02 | 0.54 | 8.25 |
|  | 1248 | 14.66 | 1.05 | 0.53 | 8.15 |
|  | 1440 | 13.94 | 1.06 | 0.53 | 7.83 |
